# A BaTiO_3_/WS_2_ composite for piezo-photocatalytic persulfate activation and ofloxacin degradation

**DOI:** 10.1038/s42004-022-00707-2

**Published:** 2022-08-10

**Authors:** Arezou Fazli, Fatemeh Zakeri, Alireza Khataee, Yasin Orooji

**Affiliations:** 1 Université Clermont Auvergne, CNRS, SIGMA Clermont, Institut de Chimie de Clermont-Ferrand, F-63000 Clermont-Ferrand, France; 2 Research Laboratory of Advanced Water and Wastewater Treatment Processes, Department of Applied Chemistry, Faculty of Chemistry, University of Tabriz, 51666-16471 Tabriz, Iran; 3 Co-Innovation Center of Efficient Processing and Utilization of Forest Resources, College of Materials Science and Engineering, Nanjing Forestry University, No. 159, Longpan Road, Nanjing, 210037 Jiangsu People’s Republic of China; 4 Department of Environmental Engineering, Gebze Technical University, 41400 Gebze, Turkey; 5 College of Geography and Environmental Sciences, Zhejiang Normal University, Jinhua, 321004 People’s Republic of China

**Keywords:** Two-dimensional materials, Pollution remediation, Catalyst synthesis

## Abstract

Piezoelectric fields can decrease the recombination rate of photogenerated electrons and holes in semiconductors and therewith increase their photocatalytic activities. Here, a BaTiO_3_/WS_2_ composite is synthesized and characterized, which combines piezoelectric BaTiO_3_ nanofibers and WS_2_ nanosheets. The piezo-photocatalytic effect of the composite on the persulfate activation is studied by monitoring Ofloxacin (OFL) degradation efficiency. Under mechanical forces, LED lamp irradiation, and the addition of 10 mM persulfate, the OFL degradation efficiency reaches ~90% within 75 min, which is higher than efficiencies obtained for individual BaTiO_3_, WS_2_, or TiO_3_, widely used photocatalysts in the field of water treatment. The boosted degradation efficiency can be ascribed to the promotion of charge carrier separation, resulting from the synergetic effect of the heterostructure and the piezoelectric field induced by the vibration. Moreover, the prepared composite displays good stability over five successive cycles of the degradation process. GC–MS analysis is used to survey the degradation pathway of OFL during the degradation process. Our results offer insight into strategies for preparing highly effective piezo-photocatalysts in the field of water purification.

## Abstract (web summary)

Piezoelectric fields can be exploited in photocatalysis to decrease the rate of photogenerated electron and hole recombination. Here, the authors demonstrate that a BaTiO_3_/WS_2_ composite is a highly effective piezo-photocatalyst for the degradation of water pollutant ofloxacin.

## Introduction

The sustainable development of human civilization is in threat by environmental pollution. Water pollution is a significant source of environmental pollution that has been aroused from the discharge of hazardous organic compounds and toxic contaminants produced in diverse industries^1^. According to the literature review, due to the wide use of antibiotics in treating human infections, a high amount of them has been released into the water. Among different antibiotics, OFL is known as a fluoroquinolone-based antibiotic that is non-biodegradable and can reach surface water^2,3^. Due to the side effect of these compounds on human health, their thorough removal from the contaminated water is an important issue addressed among researchers worldwide^2^. Sulfate radical–based advanced oxidation processes have been extensively used to degrade different ranges of water contaminants^1,2^. Wide working pH range and the production of both SO_4_^•−^ and ^•^OH make these processes feasible for degrading different ranges of water contaminants^4^. According to the literature review, different strategies such as UV irradiation, transition metal ions, and heating have been applied to activate oxidants like persulfate (PS) to produce radical sulfate anions (SO_4_^•−^)^5^.

Nevertheless, a requirement for sophisticated equipment and high energy costs are some disadvantages of these activation methods^4^. On the other hand, activation of persulfate with the assistance of heterogeneous photocatalysis has been known as a high-performance, cheap and non-toxic method for activating PS^6^. However, the broader bandgap of the photocatalysts, as well as the rapid recombination rate of a large proportion of the photogenerated electron-hole, are the essential issues that restrict the photocatalytic reactions^7^. Therefore, to improve the performance of the conventional photocatalysts, different approaches such as doping transition metals, preparing different nanostructures, and applying external electric field were used^8,9^. Recently researchers have used piezoelectric materials which can induce an electric field by applying mechanical vibration energy of ultrasonic^10^. The generated electric field can exert an opposite force on the photogenerated electrons and holes and restrict their recombination rate^7^. Therefore, integrating the inherent advantages of photocatalytic and piezoelectric materials, which results in the piezo-photocatalytic process, has been turned out to be a promising method in the field of wastewater treatment^11^. The piezoelectric effect has been shown on different single-phase ferroelectric semiconductors such as ZnO^10^, Bi_2_WO_6_^12^, MoS_2_^13^, and BaTiO_3_^14^.

On the other hand, synthesizing semiconductor heterojunctions has been confirmed to bring about many valuable properties in water treatment processes. In this regard, BaTiO_3_ has been known as a piezoelectric material that forms a built-in electric field by its spontaneous polarization^15^; however, a series of its heterostructure with different semiconductors have been reported to have more efficient photocatalytic performance for the degradation of different organic pollutants. For instance, Zhou et al.^14^ synthesized a three-layer heterojunction with BaTiO_3_, Ti_32_-oxo-cluster, and CuS. They have reported a photo-piezoelectric synergetic degradation of the target pollutants in the heterostructure mentioned above, where the tetracycline degradation was reached 100%, while it was 45% and 56% for piezoelectric and photocatalytic degradation processes, respectively. Moreover, Wu et al.^16^ proved the piezo-photocatalytic performance of the Ag_2_O/BaTiO_3_ heterostructure for the effective degradation of methyl orange.

Low dimensional materials such as Tungsten disulfide (WS_2_) possess the appropriate optical and electronic properties, which attracted the attention of researchers to use them in the field of photocatalysis^17^. Confining the carriers within the layers, providing unsaturated sulfur atoms at the edges for diverse chemical reactions, possessing an appropriate bandgap, and having a large surface area, made WS_2_ one of the excellent co-catalysts in the preparation of the heterostructures in the field of water treatment^17–19^.

The given background supports the feasibility of the synergistic photocatalysis by combining the WS_2_ nanosheets and BaTiO_3_. Therefore, we went forward to synthesize a BaTiO_3_/WS_2_ piezoelectric composite that could simultaneously use vibration and light energy. To the best of our knowledge, the BaTiO_3_/WS_2_ composite synthesis as an efficient piezo-photocatalyst for persulfate activation has not been reported previously.

The BaTiO_3_/WS_2_ piezoelectric composite was synthesized and characterized with different analyses. The synergetic effect of the piezo-photocatalytic assisted activation of persulfate was thoroughly studied. Furthermore, the photocatalytic performance of the composite was compared with TiO_2_ as the most used semiconductor in the field of photocatalytic processes. The influence of different operating parameters, like the concentration of piezo-photocatalyst and persulfate and the solution pH, was studied, and the results were interpreted in detail. For further investigation, the effect of diverse scavengers on the degradation of OFL was studied. The stability of the composite and its reusability in successive degradation processes were studied. As the final step, the underlying mechanism of OFL degradation in the piezo-photocatalytic assisted activation of PS was studied by recognizing the produced intermediates.

## Results and discussion

### Structural and morphological characterization

**Fig. 1:**
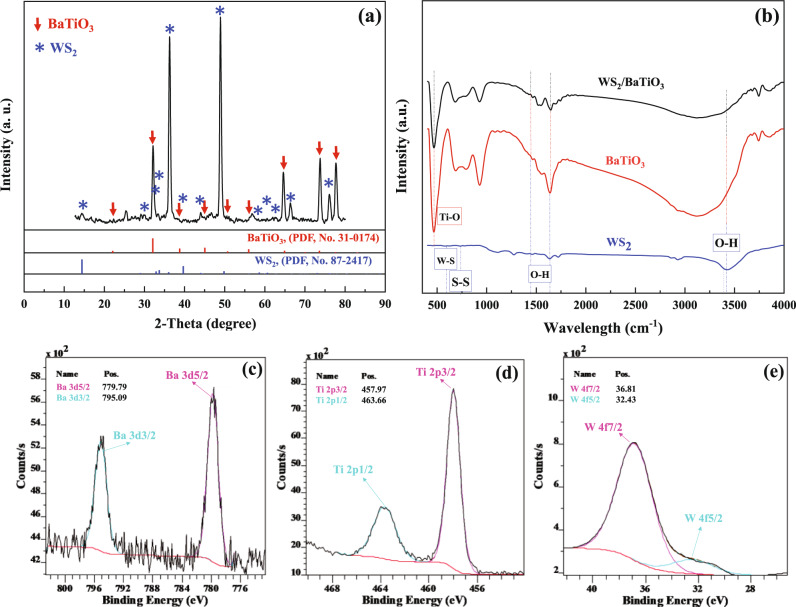
Structural characterization of the so-synthesized materials. **a** The XRD and **b** FT-IR spectra of the pure BaTiO_3_, WS_2_, and BaTiO_3_/WS_2_. High-resolution XPS spectra of **c** Ba 3d, **d** Ti 2p, and **e** W 4 f of BaTiO_3_/WS_2_ composite.

The XRD pattern of the so-synthesized BaTiO_3_/WS_2_ composite was presented in Fig. 1a. The pattern showed significant peaks at 2θ values of 14.47°, 28.97°,33.32°, 34.27°, 36.57°,40.02°, 44.8°, 48.67°, 59.57°, 62.07°, 66.42°, and 76.17 ° which were indexed to pure WS_2_ (PDF, No. 87–2417)^20^. However, the prominent peaks at 33.3°, 36.57°, and 48.7° verify the pure hexagonal structure for the resulting WS_2_ nanosheets^21^. Moreover, the main peaks appeared at 2θ values of 22.1°, 31.2°, 39°, 45°, 50.8°, 55.1°, 65.1°, 70°, 74°, and 77.2°. Accordingly, the peaks match those from the standard JCPDS data (31–0174), confirming the proper existence of BaTiO_3_ in the structure of the composite^22,23^.

Furthermore, Fig. 1b depicts the FT-IR spectrum related to BaTiO_3_, WS_2_, and composite. S − S and W − S bonds were confirmed with the peaks at 733 and 579 cm^−1^, respectively. The spectrum related to BaTiO_3_ demonstrated the presence of Ti-O at 466 cm^−1^. All the bonds mentioned above were also clearly observed in the composite of BaTiO_3_/WS_2_. In addition, the peaks in the range of 2900 cm^−1^ to 3500 cm^−1^ are related to the O-H vibration^24^. The peaks related to the 1460 and 1638 cm^−1^ correspond to the stretching deformation of hydroxyl radical^24,25^.

The valance states in the near-surface region were clarified with the XPS measurements. The high-resolution XPS spectra of Ba 3d, Ti 2p, and W 4 f of the composite are displayed in Fig. 1c–e, respectively. The peaks located at the binding energies of 457.97 and 463.66 eV correspond to Ti^4+,^^26^ in the BaTiO_3_/WS_2_ composite. As demonstrated in Fig. 1c, the specific peaks related to Ba appeared at binding energies of 779.79 and 795.09 eV^26^, which are assigned to 3d 5/2 and 3d 3/2, respectively. Furthermore, the composite shows two peaks at binding energies of 36.81 and 32.43, respectively, related to the W4f 7/2 and W4f 5/2 eV^27^. The appeared W4f peaks in the XPS spectrum of the composite are consistent with that of WS_2_, which can further confirm the presence of this phase in the composite.

**Fig. 2:**
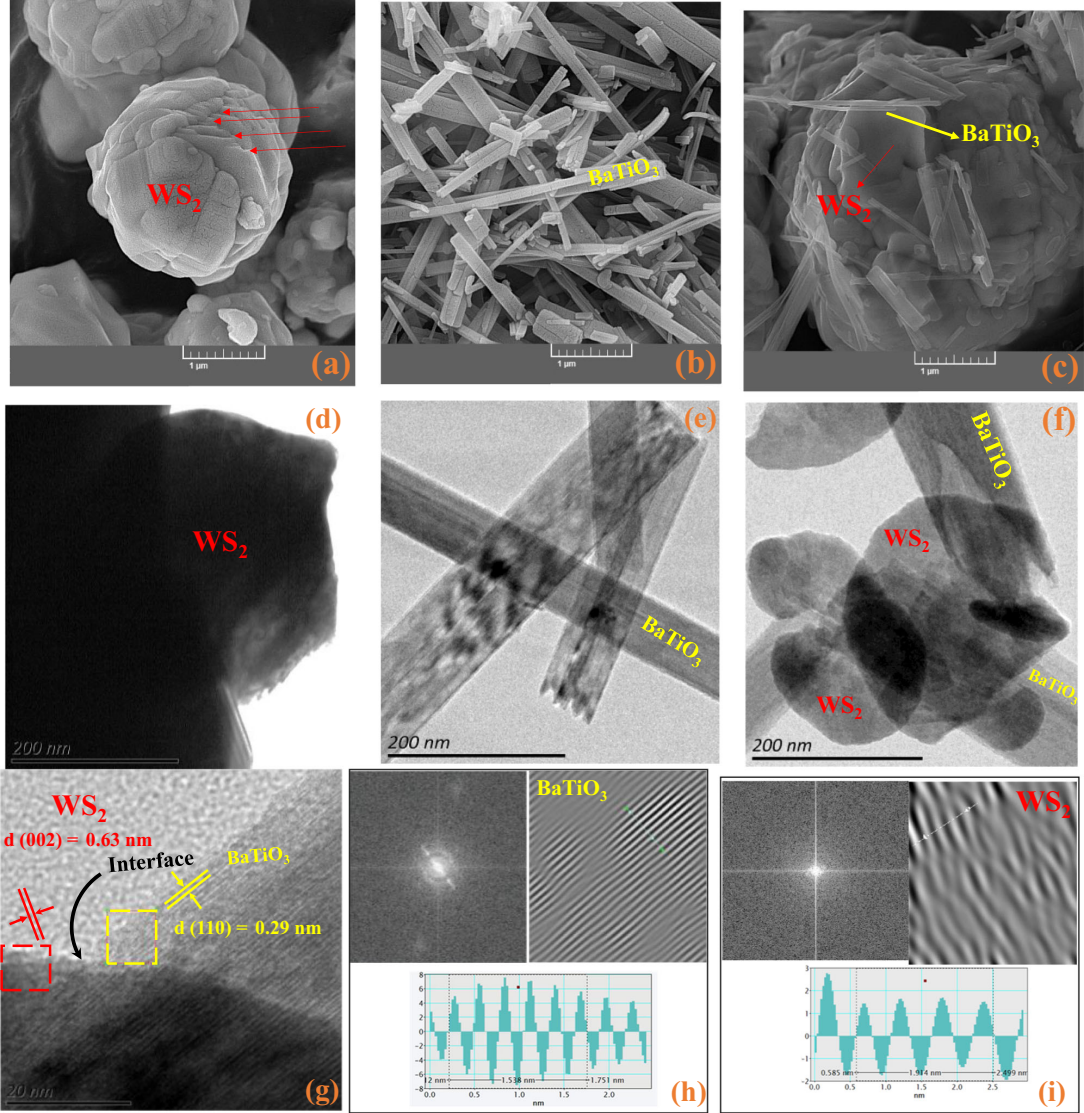
Morphological analysis of the materials. **a**–**c** SEM, and **d**–**f** TEM images of WS_2_, BaTiO_3_, and the BaTiO_3_/WS_2_ composite, respectively. **g** HR-TEM image of the BaTiO_3_/WS_2_ composite and d-spacing calculated for **h** BaTiO_3_ and **i** WS_2_ using the selected areas on HR-TEM.

Further to phase analysis, to get more information about the configuration of the above-mentioned synthesized nanomaterials, the SEM and TEM images of the pure WS_2_, BaTiO_3_, and the BaTiO_3_/WS_2_ composite were taken and the results presented in Fig. 2a–f. Figure 2a, d display the pilled WS_2_ nanosheets on each other (see Figs. S1, S2 for images). The presence of the nanosheets can be ascribed to the exfoliation of the bulk WS_2_^24^. Tizhoosh et al.^21^ have also used the same synthesis method, and they confirmed the formation of 2D WS_2_ nanosheets by exfoliating the bulk sample. On the other hand, Fig. 2b, e show the elongate and straight structure of the BaTiO_3_ nanofiber. Zhang et al.^28^ have also reported the generation of BaTiO_3_ nanofibers in the course of the two-step hydrothermal method. Furthermore, Fig. 2c, f display the SEM and TEM images of the so-synthesized composite. Given the results, the existence of the WS_2_ nanosheets and BaTiO_3_ nanofibers was confirmed, where BaTiO_3_ nanofibers covered the WS_2_ nanosheets. Further evaluation was occurred by considering the representative HR-TEM image of the BaTiO_3_/WS_2_ composite depicted in Fig. 2g. Accordingly, in the selected right side of the image, the d-spacing was calculated to be 0.29 nm which is in good agreement with the (110) plane of the BaTiO_3_ nanofibers^23^. In addition, on the left side, the d-spacing was 0.63 nm confirming the (002) plane of the WS_2_ nanosheets^29^. For more information on the calculation of d-spacing for BaTiO_3_ and WS_2_ in the selected areas, please refer to Supplementary Movie 1 and Supplementary Movie 2, respectively. The obtained results are in good coherence with the XRD pattern. In conclusion, the prepared composite consists of both WS_2_ nanosheets and BaTiO_3_ nanofibers. The interface of the WS_2_ nanosheets and the BaTiO_3_ nanofibers has been indicated in Fig. 2g.

**Fig. 3:**
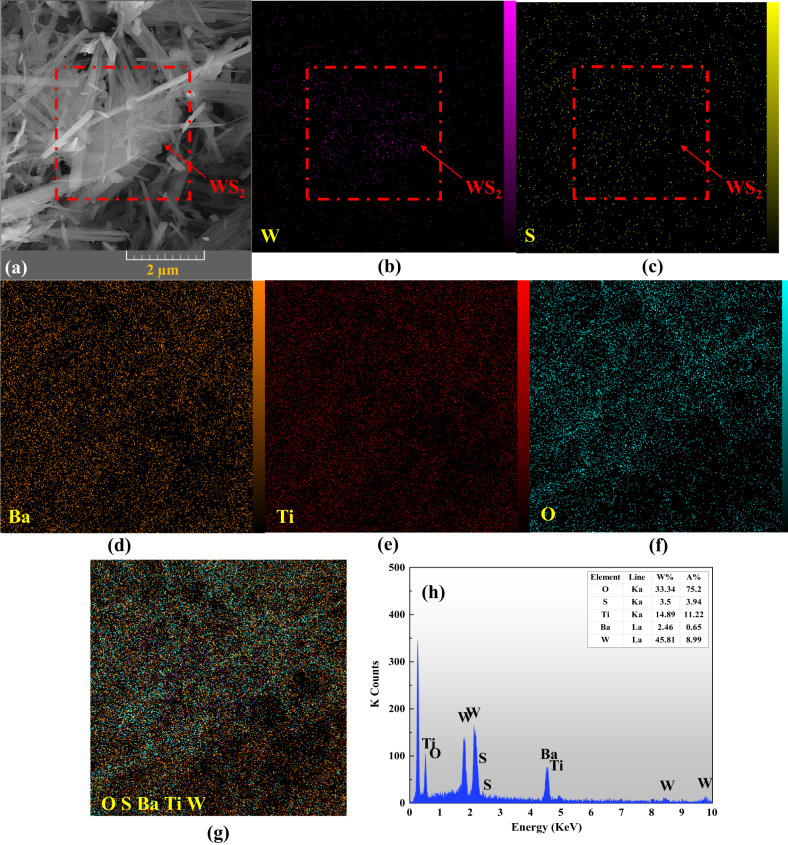
Elemental mapping graphs of the BaTiO_3_/WS_2_ composite. **a** The SEM image and **b**–**g** SEM elemental mapping of the BaTiO_3_/WS_2_ composite. **h** The EDX spectra correspond to the composite (inserted Table: quantitative results obtained for W, S, O, Ba, and Ti).

Furthermore, the SEM mapping and the EDX analysis were conducted to study the chemical composition of the composite, and the results are presented in Fig. 3a-h. Based on the X-ray emissions related to each of the elements, the purity of the BaTiO_3_/WS_2_ composite was proved by revealing the presence of Ti, Ba, W, S, and O. Furthermore, considering the indicated part on the SEM image (Fig. 3a) and the SEM-mapping of the composite (Fig. 3b, c), the existence of WS_2_ nanosheets was confirmed. Moreover, the SEM-mapping images of Ba, Ti, and O proved that the BaTiO_3_ nanofibers covered WS_2_ nanosheets.

The pore volume, as well as the BET surface area of the so-synthesized samples, were analyzed. It is worth noting that WS_2_ showed a low surface area. In this regard, Khataee et al.^24^ have also declared an almost low surface area for WS_2_ nanosheets. Considering the obtained results, the surface area and mean pore diameter of BaTiO_3_ were recorded as 10.61 m^2^g^−1^ and 18.16 nm, respectively. Nonetheless, the composite of WS_2_ and BaTiO_3_ gradually increased the surface area to 12.22 m^2^g^−1^ and 24 nm.

Moreover, the total pore volume was 0.048 cm^3^/g for BaTiO_3,_ while it was 0.074 cm^3^/g for BaTiO_3_/WS_2_ composite. For composite, the increase of surface area and the total pore volume can be related to the dispersion of BaTiO_3_ on the surface of WS_2,_ avoiding its agglomeration. Moreover, the amount of adsorbed N_2_ gas is plotted as the function of the relative pressure (Fig. S3). According to the literature review, six types of BET isotherms have been reported for different materials. Considering the obtained BET isotherms for BaTiO_3_ and BaTiO_3_/WS_2_ composite, the results accord with type IV adsorption isotherm, revealing the mesoporous characterization of BaTiO_3_ and BaTiO_3_/WS_2_ composite^30^. Selvarajan et al.^31^ have reported IV isothermal curves for BaTiO_3_ and BaTiO_3_/SnO_2_ composite.

Moreover, the UV- Vis DRS analysis was used to study the optical absorption wavelength of the so-synthesized catalysts. In this regard, the bandgap energies were calculated using the Kubelka-Monk formula and Tauc’s plot^8^. According to Fig. S4, which shows (αhν),^2^-hν curves of the prepared samples, the bandgap of BaTiO_3_, WS_2_, and BaTiO_3_/WS_2_ composite outlined with a tangent line and it found to be 3.2, 1.25, and 2.6, respectively. The results confirmed the existence of a redshift in the absorbance spectrum of the composite. This is due to the incorporation of WS_2_ into the BaTiO_3_ crystalline lattice narrowing the bandgap and consequently increasing the light absorbance in the visible zone^32^.

### Synergistic effect of the piezo-photocatalytic assisted activation of PS

**Fig. 4:**
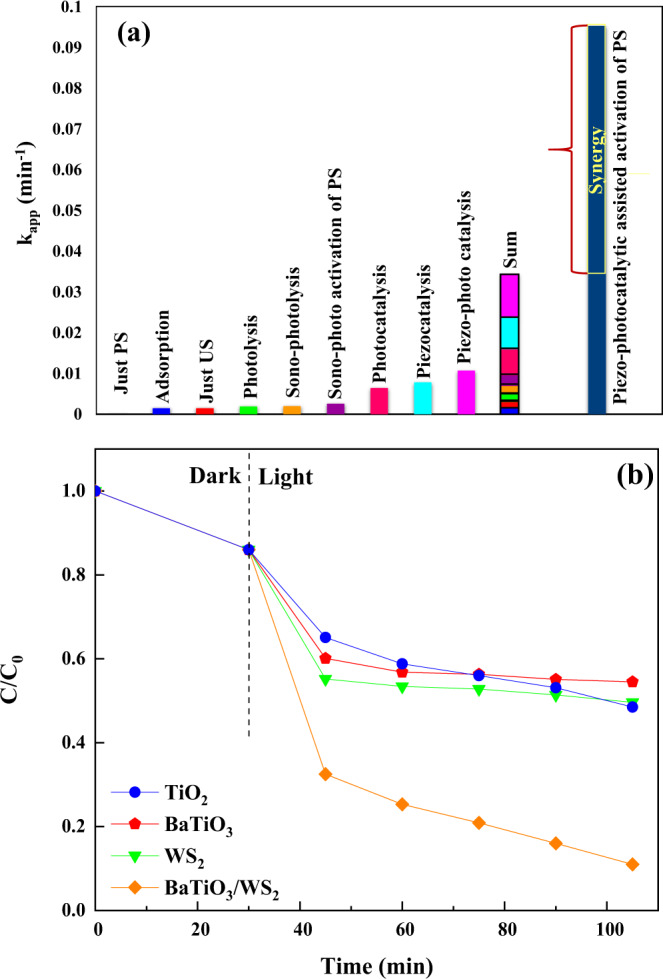
The efficiency of the BaTiO_3_/WS_2_ composite in the piezo-photocatalytic activation of persulfate. **a** Synergistic effect of the piezo-photocatalytic assisted activation of persulfate. **b** Comparison of the piezo-photocatalytic activity of the BaTiO_3_/WS_2_ composite with the pure TiO_2_, BaTiO_3_, and WS_2_ and. Experimental conditions: Catalyst concentration = 1.0 g L^−1^, [OFL] = 20 mg L^−1^, [PS] = 10 mM, pH = 6.5. The error bars were not shown, as they were smaller than the data points.

Figure S5 illustrates the performance of different processes on OFL degradation. The kinetics of the diverse processes were determined by applying the pseudo-first-order kinetic model^33^. The obtained k_app_ for different processes (Fig. 4a) shows that the individual processes are not sufficient on their own for an impressive OFL degradation. It is not the single adsorption, US, photolysis, sonophotolysis (US/light), and sono-photoactivation of PS (US/light/PS) that cause a high OFL degradation. On the other hand, confirming the results obtained from the bandgap calculation, the BaTiO_3_/WS_2_ composite could absorb the visible light and generate the necessary electron and holes to degrade OFL.

Nevertheless, the degradation efficiency of photocatalysis (light/composite) is low, which can be attributed to the high recombination rate of the photogenerated electron and holes. The contribution of external mechanical stimuli (piezocatalysis (US/composite)) could slightly raise the degradation of OFL, which in turn points out the reduction of the electron-hole recombination rate. Moreover, the results prove the low performance of sono-photoactivation of PS (US/light/PS) in the degradation of OFL, while the k_app_ of piezo-photocatalytic assisted activation of PS (US/light/composite/PS) is higher than the sum of those obtained for the individual processes.

To better describe the synergistic influence of piezo-photocatalytic activation of PS, the synergy factor and % synergy was calculated using Eq. 1 and 2. In this context, the synergy factor and % synergy was found to be 2.7 and 66.31%, respectively. These results reveal the synergistic effect for the activation of PS in the presence of BaTiO_3_/WS_2_ as the piezo photocatalyst of the process^34,35^.

1 $${{{{{\rm{Synergy}}}}}}\,{{{{{\rm{factor}}}}}}=\frac{{{{{{\rm{k}}}}}}_{{{{{\rm{app}}}}}}({\rm combined})}{{{{{{\rm{k}}}}}}_{{{{{\rm{app}}}}}}({\rm Sum}\; {\rm of}\; {\rm all}\; {\rm individual}\; {\rm processes})}$$

2 $$\% {{{{{\rm{Synergy}}}}}}=	\; \frac{{{{{{{\rm{k}}}}}}}_{{{{{{\rm{app}}}}}}}\,({{{{{\rm{combined}}}}}})-{{{{{{\rm{k}}}}}}}_{{{{{{\rm{app}}}}}}}\,({{{{{\rm{Sum}}}}}}\; {{{{{\rm{of}}}}}}\; {{{{{\rm{all}}}}}}\; {{{{{\rm{individual}}}}}}\; {{{{{\rm{processes}}}}}})}{{{{{{{\rm{k}}}}}}}_{{{{{{\rm{app}}}}}}({{{{{\rm{combined}}}}}}) }}\\ 	\times 100$$

In other words, the main reason for the synergistic effect of piezo-photocatalytic activation of PS lies on four main factors as categorized in the following. I) the irradiated photocatalysts can form the photogenerated electron-hole. II) the polarization of the composite under the ultrasound irradiation reduces the recombination rate of the electron-hole. (III) The photogenerated holes go through the oxidation of water for the production of hydroxyl radicals. Finally (V) the photogenerated electrons reduce the oxidized elements of the composite and also react with persulfate for the formation of highly active sulfate radicals^25^. A detailed discussion of this mechanism is prepared in section 3.5.

As a further evaluation, the piezo-photocatalytic efficiency of the BaTiO_3_/WS_2_ composite was also compared with pure TiO_2_, BaTiO_3_, and WS_2_ nanomaterials as the frequently used photocatalysts in the field of water treatment. According to the results of Fig. 4b, the composite of BaTiO_3_ and WS_2_ is three times as effective in removing OFL compared to pure BaTiO_3_. Furthermore, results prove that in the presence of pure WS_2_ and BaTiO_3,_ the OFL degradation stops after 10 min of irradiation, while in the presence of the BaTiO_3_/WS_2_ composite, the degradation of OFL was not stopped. These results can be attributed to the efficient charge carrier separation in the presence of WS_2_, which results in higher production of reactive radical species and increased OFL degradation^36^.

### Studying the effect of different operational conditions

**Fig. 5:**
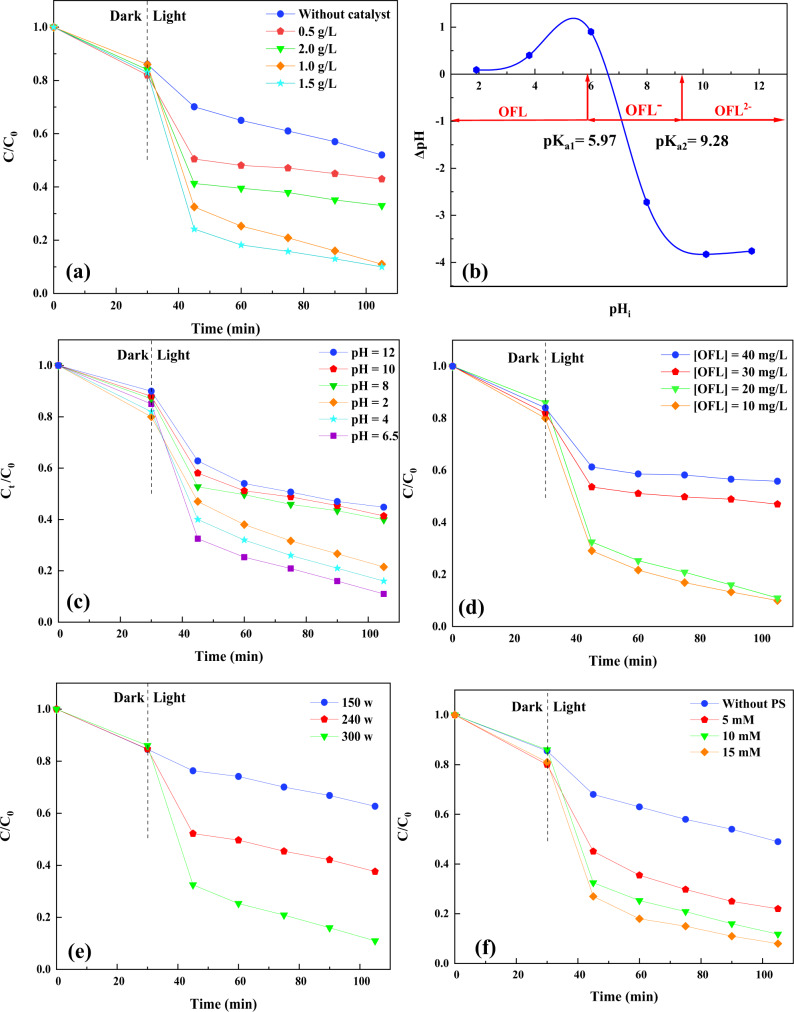
Effect of different operating parameters on the degradation efficiency of OFL. **a** Effect of catalyst concentration, **c** the effect of initiate pH, **d** various OFL concentrations, **e** bath ultrasonic power, and **f** persulfate concentration on the piezo-photocatalytic degradation of OFL. **b** pH_pzc_ of BaTiO_3_/WS_2_ nanocomposite. Experimental conditions: Catalyst concentration = 1.0 g L^−1^, [OFL] = 20 mg L^−1^, [PS] = 10 mM, pH = 6.5. The error bars were not shown, as they were smaller than the data points.

The piezo-photocatalyst concentration plays a significant role in the degradation of water contaminants^37^. A series of experiments were accomplished by using diverse concentrations of catalyst in the range of 0.5 g L^−1^ to 2.0 g L^−1^ in the piezo-photocatalytic degradation of OFL. The corresponding results are presented in Fig. 5a. Given the obtained results, the rate of OFL degradation was enhanced by increasing the catalyst concentration from 0.5 to 1.5 g L^−1^. The increased degradation efficiency can be ascribed to the presence of more active sites on the composite surface, which favors the production of different reactive oxidizing agents in the reaction media. Nonetheless, 2 g L^−1^ of the BaTiO_3_/WS_2_ composite led to a decreased degradation efficiency of OFL. According to the literature review, the aggregation of the particles and the overlapping of the active sites can be considered the primary reason for the lower yield of reactive species and the declined degradation efficiency of OFL.

Our results are in good agreement with those attained in other research^38^. For instance, in our earlier work^36^, we used WO_3_/CoFe-LDH nanocomposite as the sonocatalyst to simultaneously degrade Acid Orange 7 and Acid Blue 9. Aggregation of particles in the presence of a higher amount of the piezo-photocatalyst brought about a degradation efficiency lessening. Raising the catalyst concentration from 1.0 to 1.5 g L^−1^ brought about a slight variation in the removal efficiency of OFL, revealing the sufficiency of 1.0 g L^−1^ of the catalyst for the degradation of 89%. Hence, in the subsequent experiments, 1.0 g L^−1^ was used as the optimum value.

It is widely known that photocatalytic reactions occur on the surface of the photocatalysts^39,40^. On the other hand, the performance of the photocatalysts depends on the type of pollutants, the solution pH, and the surface’s ability to attract the water contaminants. To this end, designating the point of zero charges (pH_PZC_) for the so-synthesized catalysts is necessary to study the behavior of the catalyst and the pollutants under different pH values. The pH_pzc_ of the BaTiO_3_/WS_2_ nanocomposite is presented in Fig. 5b. Accordingly, the prepared nanocomposite showed a pH_pzc_ of 6.5. Therefore, the pH values lower than 6.5 promoted the surface of the BaTiO_3_/WS_2_ nanocomposite to be positively charged, while the surface of the catalyst was negative in the basic solutions. Besides, OFL possesses the pK_a1_ and pK_a2_ of 5.97 and 9.28, respectively^41^. Hence, the OFL molecule can be converted to its anionic form in the pH values over 5.97 (Fig. 5b).

Figure 5c shows the effect of pH (2, 4, 6.5, 8, 10, and 12) on the piezo-photocatalytic degradation of OFL. From the figure, it can be concluded that changing the solution pH from 2 to 6.5 led to a degradation efficiency of 78% to 89%, respectively. The primary reason for the promoted degradation efficiency in the lower pH ascribes two reasons. Considering the obtained pH_pzc_, the absence of repulsion force between the positively charged surface of the catalyst and the neutral OFL molecules facilitated the occurrence of the degradation reactions on the surface of the catalyst. It is believed that the acidic condition quickly stimulates the further production of reactive sulfate radicals, which then interact with the water contaminants^42,43^.

Furthermore, compared with the solution pH value of 4 and 6.5, the strongly acidic solution resulted in lower degradation efficiency for OFL. Similar results have been reported by Lebik-Elhadi et al.^42^. They studied the effect of heat and ultrasound on persulfate activation. Their findings prove that the rate constant for the thiamethoxam degradation varied from 0.173 min^−1^ to 0.153 min^−1^ in the solution pH value of 6.5 and 3, respectively^42^. The reduced degradation efficiency can be explained by the fact that the highly acidic solution results in the generation of more sulfate radicals, which can be consequently inhibited based on the reactions (2) and (3)^42^.

2 $${{{{{{\rm{SO}}}}}}}_{4}^{{{\bullet }}-}+{{{{{{\rm{SO}}}}}}}_{4}^{{{\bullet }}-}\to {{{{{{\rm{S}}}}}}}_{2}{{{{{{\rm{O}}}}}}}_{8}^{2-}$$

3 $${{{{{{\rm{SO}}}}}}}_{4}^{{{\bullet }}-}+{{{{{{\rm{S}}}}}}}_{2}{{{{{{\rm{O}}}}}}}_{8}^{2-}\to {{SO}}_{4}^{2-}+{S}_{2}{O}_{8}^{{{\bullet }}-}$$

Contrary to acidic conditions, further increase of solution pH showed less degradation performance for the target pollutant. The solution pH values over 6.5 could induce a repulsion force between the surface of the catalyst and the anionic form of OFL. However, almost 45–50% of OFL was degraded after 105 min of the degradation process, revealing the lower pH-dependence aspect of the proposed degradation process^25^. The initial solution pH of OFL was measured and reported at 6.5. Besides, the maximum degradation efficiency of OFL was achieved in the solution pH of 6.5. Therefore, the following experiments were fulfilled in the original solution pH of OFL.

According to the literature review, the environmentally-relevant concentration of most pharmaceutical pollutants has been reported to be ng. L^−1^ or lower μg. L^−1^. However, their low biodegradability and continuous release into the water resources result in their higher concentration reaching hazardous levels^44,45^. The concentration of OFL has been reported to be in the range of ng. L^−1^ to μg. L^−1 ^^46^. Nonetheless, its concentration can change from one place to another due to its different local production and dose management^47^.

The effect of a high concentration of OFL ranging from 10 to 40 mg L^−1^ was studied, and the results were presented in Fig. 5d. Accordingly, the increase of OFL concentration from 10 to 20 mg L^−1^ resulted in less reduction in the degradation efficiency. Nevertheless, a further rise of its concentration demonstrated a decreasing effect, indicating the insufficient production of the reactive oxygen radicals for the degradation of a higher amount of OFL^32^. Kamranifar et al.^48^ reported that increasing the concentration of Penicillin G had a negative effect on its photocatalytic degradation. They ascribed the reduction of Penicillin G degradation efficiency to the availability of an inadequate amount of hydroxyl radicals to degrade a higher concentration of the pollutant. One experiment with a low concentration of OFL (500 µg L^−1^) was fulfilled, and the results were presented in Fig. S6. Results reveal that in a low concentration of OFL, the degradation efficiency was almost 50% after 60 min of the process.

As one of the essential parameters in the piezo-photocatalytic degradation processes^49^, the amount of ultrasonic power was evaluated, and the results were inserted in Fig. 5e. For this purpose, experiments were done in the constant parameter conditions such as the initial solution pH of 6.5, catalyst, and PS concentration of 1.0 g L^−1^, 10 mM, respectively. Our results prove that the ultrasonic power influences the piezo-photocatalytic degradation of OFL, where the power of 300 W, 240, and 150 resulted in the OFL degradation efficiency of 89, 62.3, and 37.3%, respectively. Two different mechanisms can explain this situation. It is thought that the higher the ultrasonic power is, the faster the collapse of bubbles occurs, which in turn increases the stress and stimulates a robust piezoelectric polarization^50,51^. The existence of polarization leads to the lower recombination of the free carriers and finally brings about more involvement in the degradation of OFL^50^. Yu et al.^52^ reported similar results for the effect of ultrasonic power on the piezo-photocatalytic performance of KNbO_3_ nanosheets for the degradation of organic dyes. Since the ultrasonic power of 300 W resulted in a higher degradation efficiency in the defined experimental condition, we applied the ultrasonic power of 300 W for the subsequent tests.

Besides, one of the other significant factors that influence the degradation efficiency of the water pollutants is persulfate concentration^25^. Figure 5f displays the effect of persulfate concentration on the degradation of OFL during the piezo-photocatalytic assisted activation of PS. From the figure, it is evident that in the case of enhancing persulfate concentration up to 10 mM, the degradation efficiency of OFL was increased from 51% to 89%. Based on the literature review, the higher degradation efficiency directly relates to the higher generation of reactive species such as SO_4_^•−^ that attack the water contaminants^25^. No further enhancement in degradation efficiency was observed with a high persulfate concentration (15 mM). The slight improvement in OFL degradation (~3%) by increasing the persulfate concentration can be assigned to the self-quenching effect among the excessive radicals^53^. Therefore, the persulfate concentration of 10 mM was selected as the optimal concentration for the following experiments. To further investigate the efficiency of PS concentration of 10 mM, the decomposition of PS during the piezo-photocatalytic degradation process was studied. According to Fig. S7, almost 80% of PS was decomposed. It illustrates the high efficiency of the process for the PS activation.

Moreover, ion chromatography was used to evaluate the concentration of sulfate ions in the treated water. The concentration of sulfate ions was found to be ~477 mg L^−1^ which is lower than the standard concentration (500 mg L^−1^) reported by the World Health Organization^54^. Obtained results reveal the appropriate applicability of the selected PS concentration to prepare non-toxic treated water.

It is worth noting that the degradation efficiency of diverse types of organic pollutants like dyes and pesticides was also monitored to evaluate the degradation performance of the piezo-photocatalytic process in the presence of PS. Methylene blue (MB) is one of the azo dyes which is used in industries and results in different diseases like fever and skin desquamation in humans^55,56^. Moreover, it can affect the ecosystem by covering the surface of the water and decreasing the rate of photosynthesis of the water plant^55^. Besides, paraquat (PQ) is used as a fast-acting and non-selective pesticide that can control the growth of weeds in agricultural products^57^. Toxic of paraquat can be harmful to human beings and animals. Therefore, we selected these compounds as representatives of other types of water contaminants and followed their degradation efficiency during the piezo-photocatalytic assisted activation of PS. Based on Fig. S8, the degradation efficiency of MB and PQ was found to be 95.3 and 98.5%, respectively. The results reveal that the piezo-photo catalytic assisted activation of PS in the presence of BaTiO_3_/WS_2_ has a high performance on the degradation of different types of organic contaminants.

### Reusability and stability

**Fig. 6:**
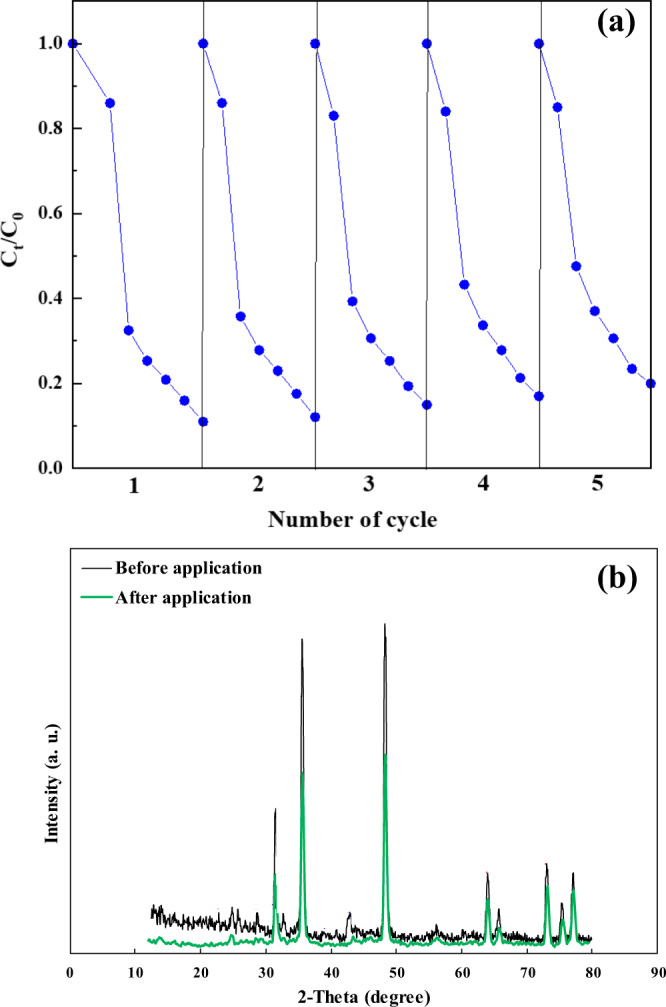
The reusability and stability of the BaTiO_3_/WS_2_ composite. **a** The piezo-photocatalytic degradation efficiency of OFL and **b** XRD patterns of the BaTiO_3_/WS_2_ composite before and after five successive cycles. Experimental conditions: Catalyst concentration = 1.0 g L^−1^, [OFL] = 20 mg L^−1^, [PS] = 10 mM, pH = 6.5.

In terms of cost-effectiveness, the stability and reusability of catalysts are the main factors in the heterogeneous water treatment processes^36^. Hence, the reuse performance and durability of the BaTiO_3_/WS_2_ composite were examined in five times successive repeating OFL degradation experiments. The used catalyst was centrifuged, dried at 50^o^ C, and used in the following experiment. The degradation efficiency of OFL after five cycles of the degradation processes is reported in Fig. 6a. Accordingly, the degradation efficiency of OFL was maintained at a high level (80%) even after five cycles of the degradation process, indicating the stability and reusability of the BaTiO_3_/WS_2_ composite.

Moreover, Fig. 6b shows the XRD pattern of the used and fresh BaTiO_3_/WS_2_ composite. The XRD patterns of both samples showed the main peaks at almost the same 2theta position, which proved that the crystallinity and composition of the catalyst were not changed. The concentration of the leaching elements was assessed by using Atomic Absorption Spectroscopy. The results revealed that W, Ti, and Ba concentration was 1 µg L^−1^, 0.07, and 0.067 mg L^−1^. According to the world health organization, the standard concentration of W, Ti, and Ba in the drinking water is 1.9 µg L^−1^, 0.1 mg L^−1^, and 1.3 mg L^−1^, respectively. Therefore, the concentration of the leaching elements at the solution pH of 6.5 was lower than the standard concentration of them in the drinking water.

### Catalytic mechanism

**Fig. 7:**
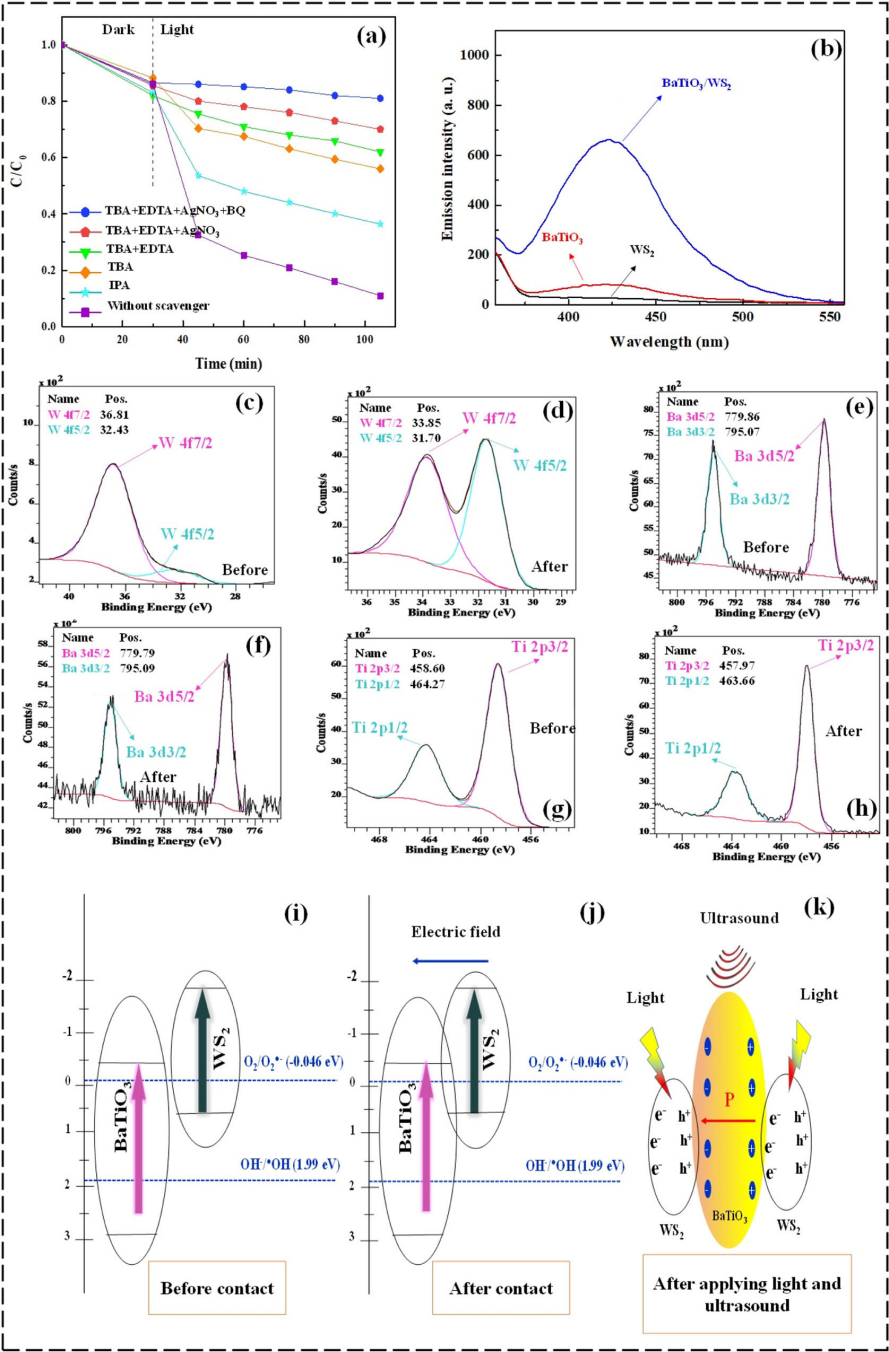
Evaluating the produced ROSs and piezo-photocatalytic mechanism. **a** The effect of diverse scavengers on the piezo-photocatalytic degradation of OFL in the presence of PS, **b** Fluorescence spectra of the materials in the presence of AgNO_3_, **c**–**h** high-resolution XPS spectra of W, Ba, and Ti before and after contact of WS_2_ and BaTiO_3_, and **i**–**k** the proposed schematic mechanism for the charge-carrier transference of the BaTiO_3_/WS_2_ composite. Experimental condition: Catalyst concentration = 1.0 g L^−1^, [OFL] = 20 mg L^−1^, pH = 6.5, [Scavengers] = 5 mmol L^−1^. The error bars were not shown, as they were smaller than the data points.

The piezophototronic effect-induced photoredox reactions were clarified by assessing the contribution of reactive oxygen species (ROSs) in the decomposition of OFL. In this regard, the role of produced active oxidizing agents such as ^•^O_2_, ^•^OH, h^+^, and e^−^ during the piezo-photocatalytic assisted activation of PS over BaTiO_3_/WS_2_ composite was investigated by using various ROSs scavengers. Based on Fig. 7a, iso-butanol (IBA), as a well-known ^•^OH scavenger, led to the variation of OFL degradation from ~90% to ~60%. It can be attributed to the contribution of ^•^OH scavenger. Moreover, to recognize the existence and contribution of sulfate radicals (SO_4_^•−^) in the course of the degradation process, tert-butanol (TBA) was used. TBA can serve as the inhibitor for both ^•^OH and SO_4_^•−^. Therefore, the observed further inhibition (~20%) corresponds to the contribution of SO_4_^•−^ in the degradation of OFL. Ethylenediaminetetraacetic acid (EDTA) is a scavenger that traps the photogenerated holes; however, considering the NIST Kinetics Database, it can also go through the competing reactions with ^•^OH. For this reason, TBA was added to the reaction media to inhibit the ^•^OH and leave EDTA free to react only with photogenerated holes. To study the contribution of electrons in the degradation of OFL, AgNO_3_ was applied. Similarly, the experiment was fulfilled in the presence of TBA and EDTA to avoid the reaction of AgNO_3_ with hydroxyl radicals and photogenerated holes. This showed further (8%) inhibition of OFL degradation, which attributes to the existence of electrons. On the other hand, benzoquinone (BQ) has been reported as the ^•^O_2_ scavenger^50^. Nonetheless, regarding NIST Kinetics Database, the rate constants of BQ with ^•^OH, SO_4_^•−^, and ^•^O_2_ were reported to be 1.2 × 10^9^, 1.2 × 10^9^, and 9 × 10^8 ^M^−1^ S^−1 ^^58^. In case of not using other scavengers to quench ^•^OH, SO_4_^•−^, and possible electron-hole, BQ cannot be ideal for studying the contribution of ^•^O_2_. Consequently, in the presence of TBA, EDTA, and AgNO_3,_ benzoquinone showed 11% more inhibition, which can be related to the presence of superoxide radicals in the reaction media.

Considering the gained results, the photogenerated h^+^ and e^−^ of the BaTiO_3_/WS_2_ composite have an adequate redox potential to prepare ^•^OH and O_2_^•−^. The XPS valance band spectra were used to determine the conduction band (CB) and valance band (VB) potential of the individual semiconductors. The XPS valance spectra of the WS_2_ and BaTiO_3_ are presented in Fig. S9a, b. Consequently, the VB edge potentials of the so-synthesized BaTiO_3_ and WS_2_ were found to be 2.8 and 0.68 eV, respectively. The obtained values for the VB edge were in good accordance with the ones reported for the few-layer WS_2_ and BaTiO_3_ nanofibers^59,60^. As the bandgap energy has already been calculated from the DRS results, the CB of BaTiO_3_ and WS_2_ was also calculated using Eq. 3.

3 $${{{{{\rm{E}}}}}}({{{{{\rm{VB}}}}}}) ={{{{{\rm{E}}}}}}({{{{{\rm{CB}}}}}})-{{{{{\rm{Eg}}}}}}$$

Accordingly, the CB was calculated to be −0.4 and −1.93 eV for BaTiO_3_ and WS_2_, respectively, which accords with the results reported for the same materials used in other research papers^59,60^. Taking account of the schematic figure inserted in Fig. 7i, before contacting the present electrons in the CB of each component were potential enough to reduce oxygen and form superoxide and hydroxyl radicals, whereas the VB of WS_2_ was not suitable to oxidize water to ^•^OH. Therefore, complementary experiments were fulfilled to track the activity of piezo photogenerated holes in the VB of each BaTiO_3_, WS_2_, and BaTiO_3_/WS_2_ composite. For this purpose, the production of ^•^OH in piezo-photocatalytic reactions (without adding PS) was evaluated by applying TA in the presence of an electron scavenger. In Fig. 7b, it is clear that WS2 did not show any fluorescence response at 423 nm in the absence of piezo photogenerated electrons. The main reason is the incompetent holes in VB of WS_2_ to induce oxidation and produce ^•^OH, which can finally react with TA to produce a fluorescent **2-**hydroxyterephthalic acid (HTA). However, fluorescent spectra were observed for BaTiO_3_ and composite, which was more intense for composite. These findings further imply that ^•^OH was produced over BaTiO_3_ and BaTiO_3_/WS_2_ composite even in the absence of piezo photogenerated electrons, suggesting the contribution of holes in oxidizing reactions. Moreover, the increased fluorescence intensity in the presence of BaTiO_3_/WS_2_ composite reveals the higher production of piezo photogenerated electron/hole and the appropriate charge carrier movement to reduce their recombination rate^8^. Zhou et al.^61^ have also used a terephthalic acid solution to study the production of ^•^OH radical in the course of the piezo-photocatalytic reaction. They reported that more amount of hydroxyl radicals were generated over BiOX, BaTiO_3_, and BiOX/BaTiO_3_ composite, while the fluorescence intensity was higher for the composite^61^.

For further investigation, the high-resolution XPS spectra of W 4 f, Ba 3d, and Ti 2p in samples of WS_2_, BaTiO_3_, and their composite were recorded and compared before and after contact. From the spectra, it is clear that the binding energy of W 4 f was shifted from the lower energy in the pure WS_2_ to higher positions in the BaTiO_3_/WS_2_ composite. Nonetheless, the prominent peaks of Ba 3d and Ti 2p in the pure BaTiO_3_ appeared in the higher binding energies, while the same peaks were moved to the lower energies in the BaTiO_3_/WS_2_ composite. The opposite shift can be related to the existence of an effective interfacial contact of BaTiO_3_ and WS_2_, which in turn results in the formation of an electric field pointing from WS_2_ to BaTiO_3_ and consequently equilibrium of the Fermi levels^8^. Moreover, based on the literature review, piezoelectric materials such as BaTiO_3_ can produce free radicals and create a catalytic performance in water through its polarization^62^. Hence, the high piezo-photocatalytic performance can be due to the appropriate charge carrier separation coming from I) an electric field between the composite components (Fig. 7j) and II) the ferroelectricity property of BaTiO_3_ in BaTiO_3_/WS_2_ composite leading to the polarization when exposed to ultrasonic vibration (Fig. 7k). This polarization can bring about the generation of superoxide and hydroxide radicals in water^23^.

### The degradation mechanism of OFL

**Fig. 8:**
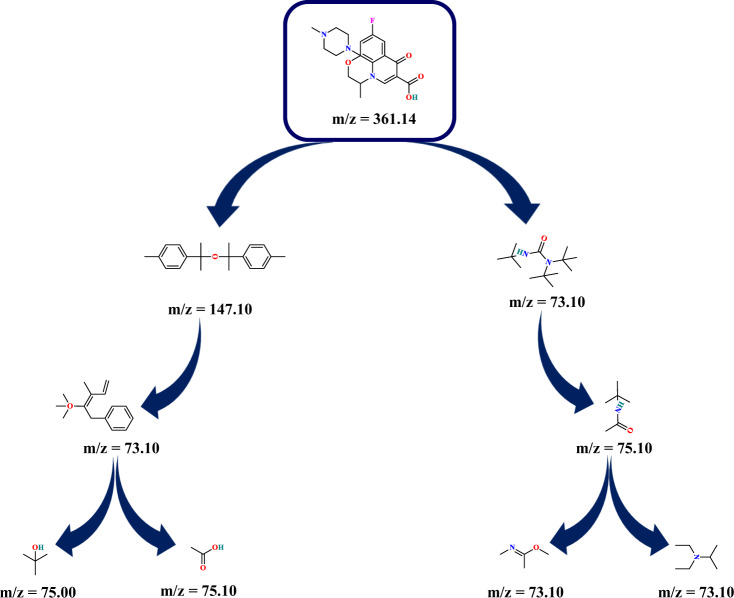
Degradation mechanism of OFL. Proposed reaction pathways for OFL based on the determined by-products.

The produced intermediates during the OFL degradation via piezo-photocatalytic assisted activation of PS were detected by GC–MS analysis. Diethyl ether and N, O-bis-(trimethylsilyl)-acetamide were used to extract intermediates existing in the sample. Therefore, the main chromatogram and the characterizations of the intermediates were reported in Fig. S10 and Table. S1, respectively. Eight primary intermediates produced during the degradation process proved the breakage of rings and the , , , and  bonds of OFL. According to these by-products, the degradation pathway has been proposed in Fig. 8. The reactive radical species (SO_4_^•−^ and ^•^OH) attacked the OFL molecules, dissociated the rings, and formed other by-products such as carboxylic acids and amides. According to the literature review, the generation of amides and carboxylic acids stimulates the induce mineralization of organic contaminants, which is favorable in wastewater treatment^32^.

## Conclusion

The BaTiO_3_/WS_2_ composite was successfully synthesized via a two-step hydrothermal method. XRD, XPS, FT-IR, EDX, SEM, TEM, HRTEM, BET, and DRS analyses were used to analyze the so-synthesized materials. The XRD, and HRTEM images exhibited the appropriate co-existence of WS_2_ nanosheets and BaTiO_3_ nanofibers in the composite. Moreover, the prepared composite showed a high piezo-photocatalytic performance in activating persulfate to sulfate radicals. The experiments regarding the free radical trapping revealed the main contribution of Sulfate, hydroxyl and superoxide radicals in degradation reactions.

Moreover, the high-resolution XPS spectra of W 4 f, Ba 3d, and Ti 2p before and after the contact of WS_2_ and BaTiO_3_ showed the electron flow from WS_2_ to BaTiO_3_ after their physical contact. Considering the fluorescence experiments in the presence of terephthalic acid, the BaTiO_3_/WS_2_ composite could use a visible light source and use the piezoelectric source to effectively prevent recombination of photogenerated charge carriers and accelerate the production of hydroxyl radicals. This work implies designing two-components composites; one component with the piezo-photocatalytic activity and the other with the thin layered structure to facilitate the charge carrier movement and enhance their separation. In conclusion, the BaTiO_3_/WS_2_ composite can be introduced as a visible-light responsive and effective piezo-photocatalyst which can activate persulfate for degradation of different water contaminants.

## Methods

### Applied chemical compounds

Ba(OH)_2._ 8H_2_O ( >98%), TiO_2_ (99.9%), NaOH (≥98%), WS_2_ (99%), Dimethylformamide (DMF, 97%), Ethanol (C_2_H_5_OH, 96%), tert-butanol (C_4_H_10_O, ≥99.5%), N, O-bis-(trimethylsilyl)-acetamide, **1,4-**benzoquinone (C_6_H_4_O_2_, 99%), and Na_2_S_2_O_8_ (≥99%) were provided from Sigma Aldrich (USA). Diethyl ether (C_4_H_10_O, 99%), Iso-butanol (C_4_H_10_O, 99.5%), Ethylenediaminetetraacetic acid (EDTA, ≥99%), and AgNO_3_ (≥99%) were provided by Merck (Germany). Ofloxacin was obtained from Rouz Darou Laboratory (Iran). All the used chemicals were analytically pure and were used as they were purchased without further purification. Moreover, deionized water was used for the experiments and the synthesis of the nanomaterials. X-ray diffraction (XRD, on a D8 Advance X-ray diffractometer from Bruker, Germany) with a Cu Kα radiation: 0.15406 nm at an accelerating current of 40 mA and voltage of 45 kV was used to determine the crystalline phase of the prepared nanomaterials. The scanning electron microscopy (SEM) images were achieved on a Tescan Mira3 microscope (Czech Republic) equipped with an energy dispersive X-ray (EDX) spectrometer. The High-resolution transmission electron microscopy (HRTEM) was carried out by JEM-2100, Jeol (Japan) The ultraviolet-visible diffuse reflectance absorption spectra (DRS) were recorded on an Analytik Jena spectrophotometer (S 250, Germany). FT-IR spectrum has been recorded by a Bruker Tensor-27 (Germany) spectrophotometer. Moreover, the specific surface area of the so-synthesized catalysts was studied using N_2_ adsorption/desorption isotherms at 77 K applying the Brunauer–Emmett–Teller (BET) method using 3 Flex instruments (Micromeritics, USA). Finally, X-ray photoelectron spectroscopy (XPS) was carried out using a Thermo scientific photoelectron spectrometer (Kratos AXIS UltraDLD, UK).

### Synthesis of 2D WS_2_ nanosheets

An ultrasonic-assisted method was used to prepare the WS_2_ nanosheets from the bulk WS_2_^21^. In this context, 0.5 g of pure WS_2_ powder was added to 50 mL of DMF and sonicated for three hours using an ultrasonic bath (300 W). The exfoliated WS_2_ particles were separated by centrifuging at 1000 rpm for 5 min. The gained precipitate was washed 2–3 times with ethanol and distilled water and dried for 12 h.

### Synthesis of BaTiO_3_ nanofibers

A two-step hydrothermal^63^ method was used for the preparation of BaTiO_3_ nanofibers. Briefly, 1.5 g titanium oxide was dissolved in 70 ml of NaOH solution (10 M) and stirred for 2 h to form a homogeneous suspension. Afterward, the solution was transferred to a 100 ml Teflon-lined autoclave and heated at 210 °C for 24 h. The resulting precipitate was filtered, washed with distilled water, and soaked in HCl 0.2 M for 4 hours. Accordingly, the H_2_Ti_3_O_7_ nanofibers were dried and collected to synthesize BaTiO_3_ nanofibers. For this purpose, the mixture of 0.150 g of so-synthesized H_2_Ti_3_O_7_ nanofibers and 70 mL of Ba(OH)_2_ ∙ 8H_2_O solution were sonicated for 15 min. Then, the second hydrothermal reaction occurred at 210 °C for 24 h in a 100 ml Teflon-lined autoclave. The obtained precipitates were briefly soaked in hydrochloric acid solution (0.2 M), washed thoroughly with distilled water, and dried at 80 °C.

### Synthesis of BaTiO_3_/WS_2_ composite

The synthesis method of BaTiO_3_ was adopted to prepare the BaTiO_3_/WS_2_ composite. However, according to Fig. S11, the predetermined amount of WS_2_ nanosheets (15 wt%) was added to 70 mL Ba(OH)_2_ ∙ 8H_2_O (0.008 M) and sonicated for 10 min. Then, 0.150 g of so-synthesized H_2_Ti_3_O_7_ nanofibers were added to the mixture and sonicated for more than 10 minutes. The obtained mixture was transferred to a 100 ml Teflon-lined autoclave, and the hydrothermal reactions occurred at 210 °C for 24 h. The resulting gray precipitate was washed several times with the above-described method and dried at 80 °C.

### Piezo-photocatalytic experiments

The piezo-photocatalytic activity of the so-synthesized samples for persulfate activation was carried out at room temperature and ambient pressure. A yellow LED light with a wavelength of approximately 580 nm, and an ultrasonic bath (EP S3, Sonica, 40 kHz, 300 W, Italy) were used as the system’s light and vibration energy source. It is worth noting that the ultrasonic bath is equipped with a microprocessor that keeps the temperature at 25°C. A chemical actinometric method using potassium ferrioxalate was used to measure the radiant flux of the lamp^64^. Accordingly, the lamp’s incident photon flux was found to be 4.2 × 10^−6^ einstein s^−1^. Furthermore, by applying the calorimetric method^65^, the actual power delivered from the sonication was different from the output power of the ultrasound generator. In this regard, a thermocouple was used to record the temperature (T) variation resulting from the mechanical energy of the ultrasonic generator against time (t). Therefore, the real ultrasonic power delivering the system was calculated by power = m Cp (dT/dt), where m, and Cp are water’s mass and heat capacity (4.187 kJ kg^−1^K^−1^), respectively^65^. Hence, the actual power of sonication was 172 W.

100 mL of OFL was added to the Erlenmeyer flask (250 mL) containing a specific amount of photocatalysts for each run. HCl and NaOH were used to adjust the initial pH of the OFL solution. Before starting the degradation process, the mixture was stirred for 30 min to get adsorption/desorption equilibrium in the dark. Then a specific amount of persulfate was added to the solution, and the sonocatalytic degradation process was started in the presence of ultrasound waves and visible light. 3 mL of the treated sample was collected from the reactor at different time intervals. To avoid the possible oxidation reactions, 1 mL of the methanol (with no absorption at 290 nm) was added to the withdrawn samples. After filtering the samples, the remained concentration of the OFL was analyzed using a UV spectrophotometer at the maximum wavelength of 290 nm. The corresponding degradation ratio ($\frac{{C}_{t}}{{C}_{0}}$) of OFL was achieved through the ratio of obtained absorbance for the sample withdrawn at times 0 and t. The error bars representing ± one standard deviation were reported from the mean of two measurements. The persulfate decomposition was followed by the iodide method described previously^66^.

To assess the possibility of absorbing the generated by-products at 290 nm, the collected samples before and after the degradation process were analyzed using high-performance liquid chromatography (HPLC, Waters 2695, USA). For this purpose, a mobile phase with methanol/formic acid composition in ultrapure water (15:85, v/v) was used. The flow rate was set at 1.0 mL∙min^−1^, and the wavelength of the UV–vis detector was fixed at 288 nm. It was observed that at 290 nm, all the peak was related to the OFL.

The solution pH variation by adding the PS was studied before starting and during the degradation process. The results are presented in Table. S2 at the supplementary data. Accordingly, after adding PS and during the degradation process, the pH of the solution was gradually decreased.

The point of zero charges (pH_pzc_) for the synthesized catalysts was measured using a method that has been described in our previously published paper^36^. The reactive oxidizing species in the degradation reaction were detected using different scavengers. The produced hydroxyl radicals were tracked using a terephthalic acid (TA) as a probe agent. Nonfluorscence TA reacts with the available ^•^OH, which yields **2-**hydroxyterephthalic acid which is fluorescent and can have an emission peak at 425 nm with the excitation at 380 nm. Therefore, the samples were prepared, and the concentration of 2-hydroxyterphthalic was monitored by a Varian Cary Eclipse, USA fluorescence spectrophotometer. The produced intermediates were followed using the gas chromatography-mass spectrometry (GC–MS) analysis. For this purpose, an Agilent 6890 system and an Agilent 5973 mass instrument (Agilent Technologies, Canada) were used.

## Supplementary information

Supplementary Information

Description of Additional Supplementary Files

Supplementary Movie 1

Supplementary Movie 2

## Data Availability

The authors declare that all the data supporting the conclusion of the present study are available within the paper and supporting information and also from the corresponding author upon reasonable request.
