# Supplementary material for: A BaTiO3/WS2 composite for piezo-photocatalytic persulfate activation and ofloxacin degradation
Source: Commun Chem. 2022 Aug 10;5:95. doi: 10.1038/s42004-022-00707-2 (PMC9814951; doi:10.1038/s42004-022-00707-2)
Supplement: Supplementary file 1 — Supplementary Information [file 42004_2022_707_MOESM1_ESM.pdf]

Supporting information

A $BaTiO_3/WS_2$ composite for piezo-photocatalytic persulfate activation and ofloxacin degradation

Arezou Fazli[1,2,†], Fatemeh Zakeri[3,2,†], Alireza Khataee[2,4*], Yasin Orooji[3,*]

[1] *Universit´e Clermont Auvergne, CNRS, SIGMA Clermont, Institut de Chimie de Clermont-Ferrand, F-63000 Clermont-Ferrand, France*

[2] *Research Laboratory of Advanced Water and Wastewater Treatment Processes, Department of Applied Chemistry, Faculty of Chemistry, University of Tabriz, 51666-16471, Tabriz, Iran*

[3] *College of Geography and Environmental Sciences, Zhejiang Normal University, Jinhua, 321004, China*

[4] *Department of Environmental Engineering, Gebze Technical University, 41400 Gebze, Turkey*

† These authors contributed equally.

* Corresponding authors:

a_khataee@tabrizu.ac.ir (A. Khataee)

orooji@zjnu.edu.cn (Y. Orooji)

Supplementary figures

Fig. S1. SEM images of the materials.

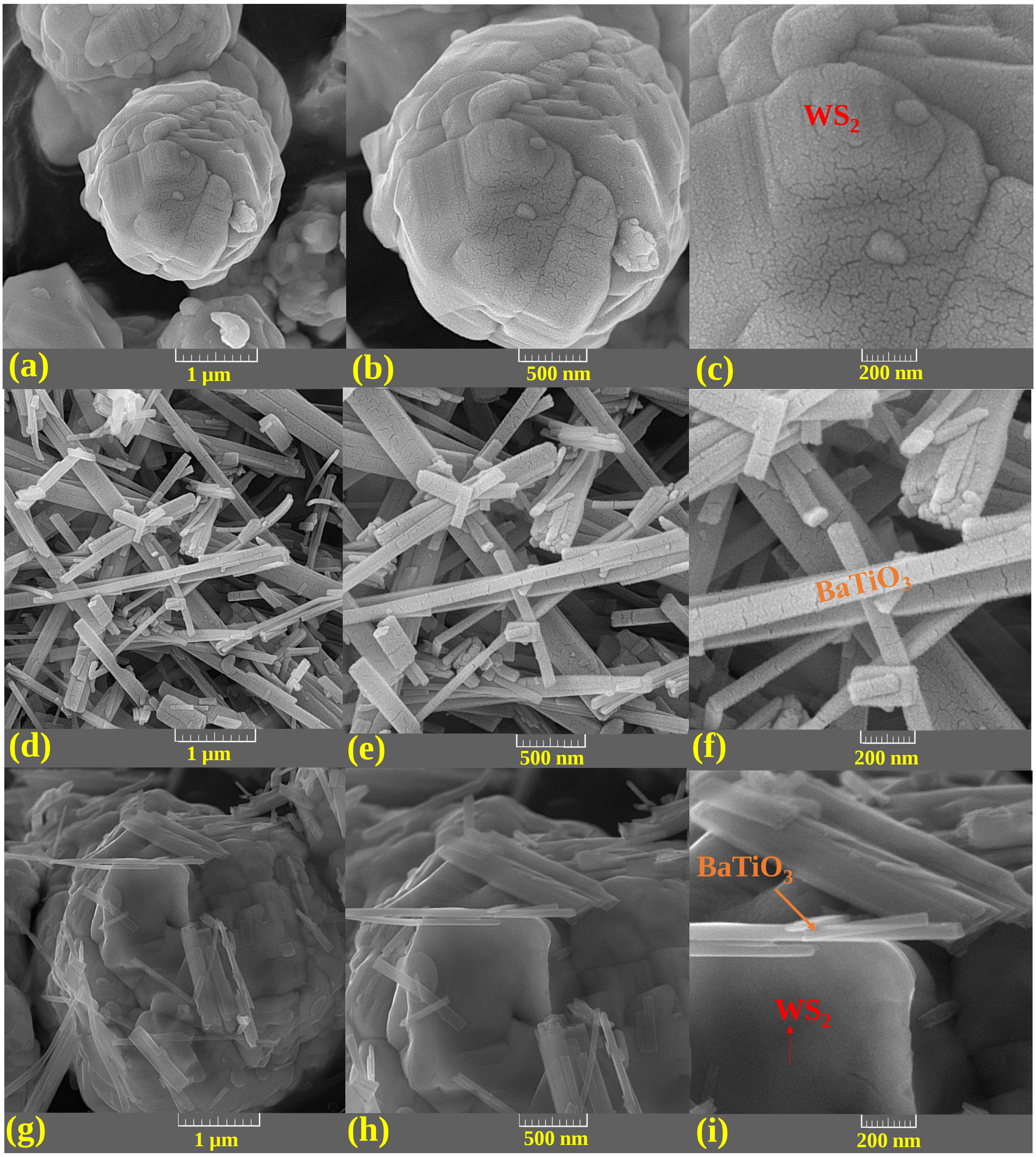

(a-c) pure WS_2, **(d-f)** pure $BaTiO_3$, and **(g-i)** $BaTiO_3/WS_2$ composite.

Fig. S2. TEM images of the materials.

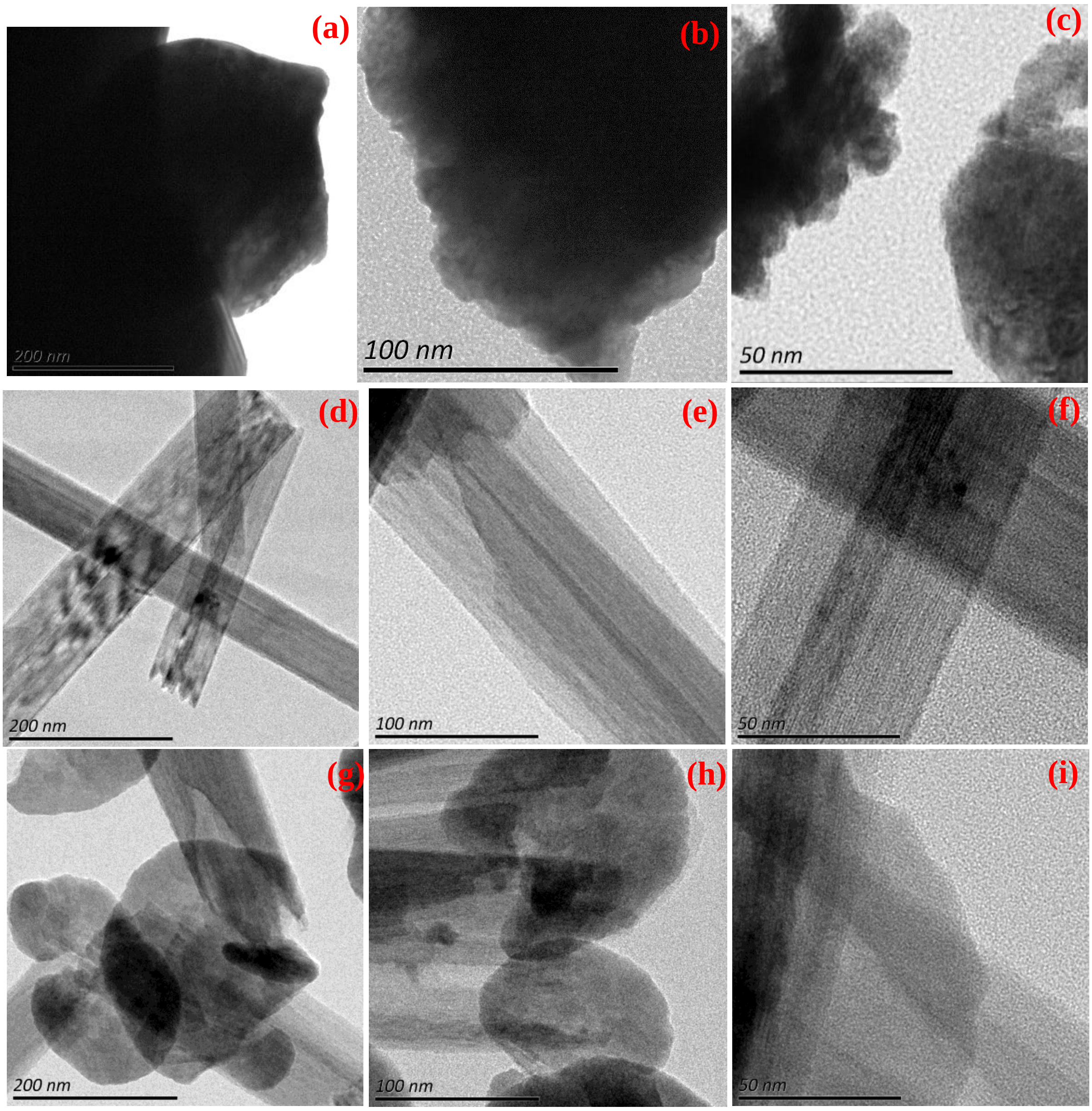

(a-c) pure WS_2, **(d-f)** pure $BaTiO_3$, and **(g-i)** $BaTiO_3/WS_2$ composite.

Fig. S3. N_2 adsorption/desorption isotherms of the synthesized materials.

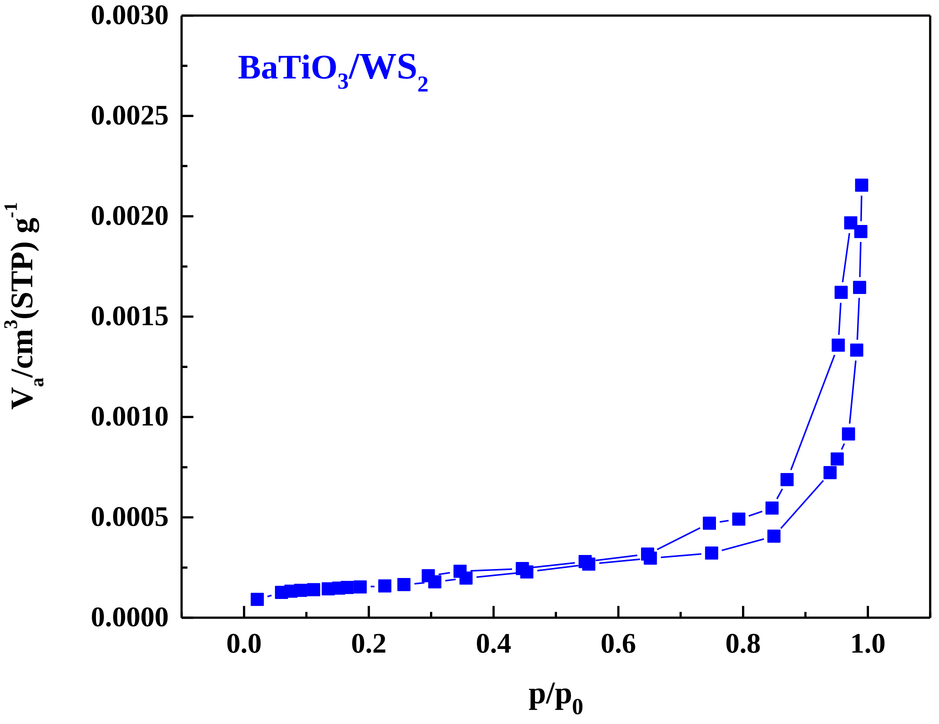

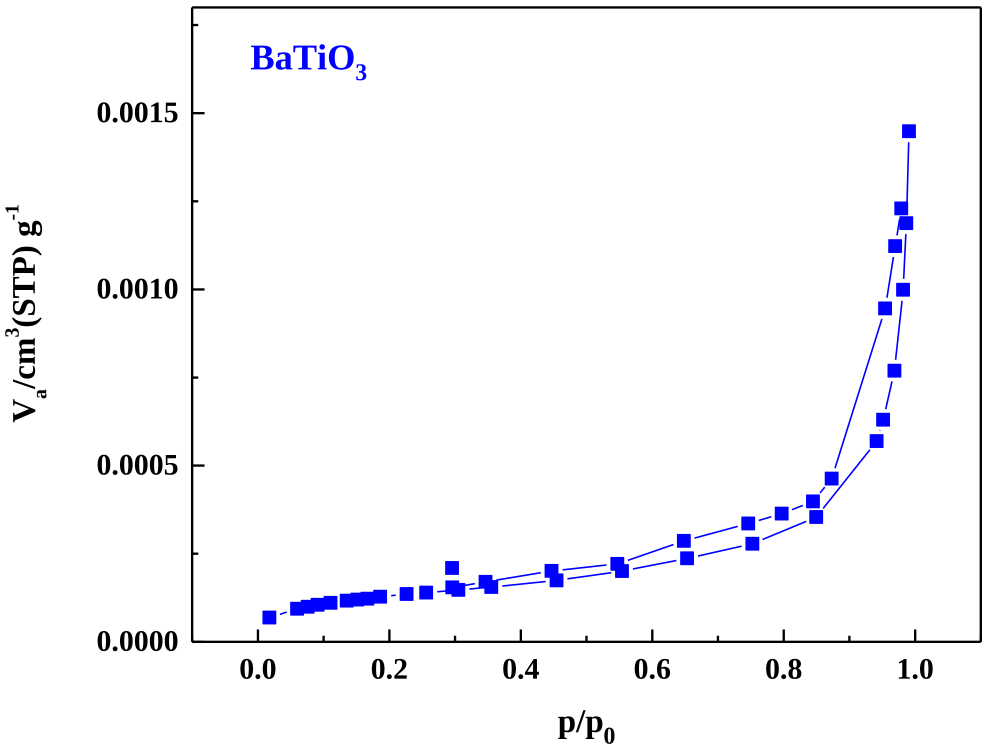

Fig. S4. The $(\alpha h\upsilon)^2$-hυ curves for WS_2, $BaTiO_3$, and $BaTiO_3/WS_2$ composite.

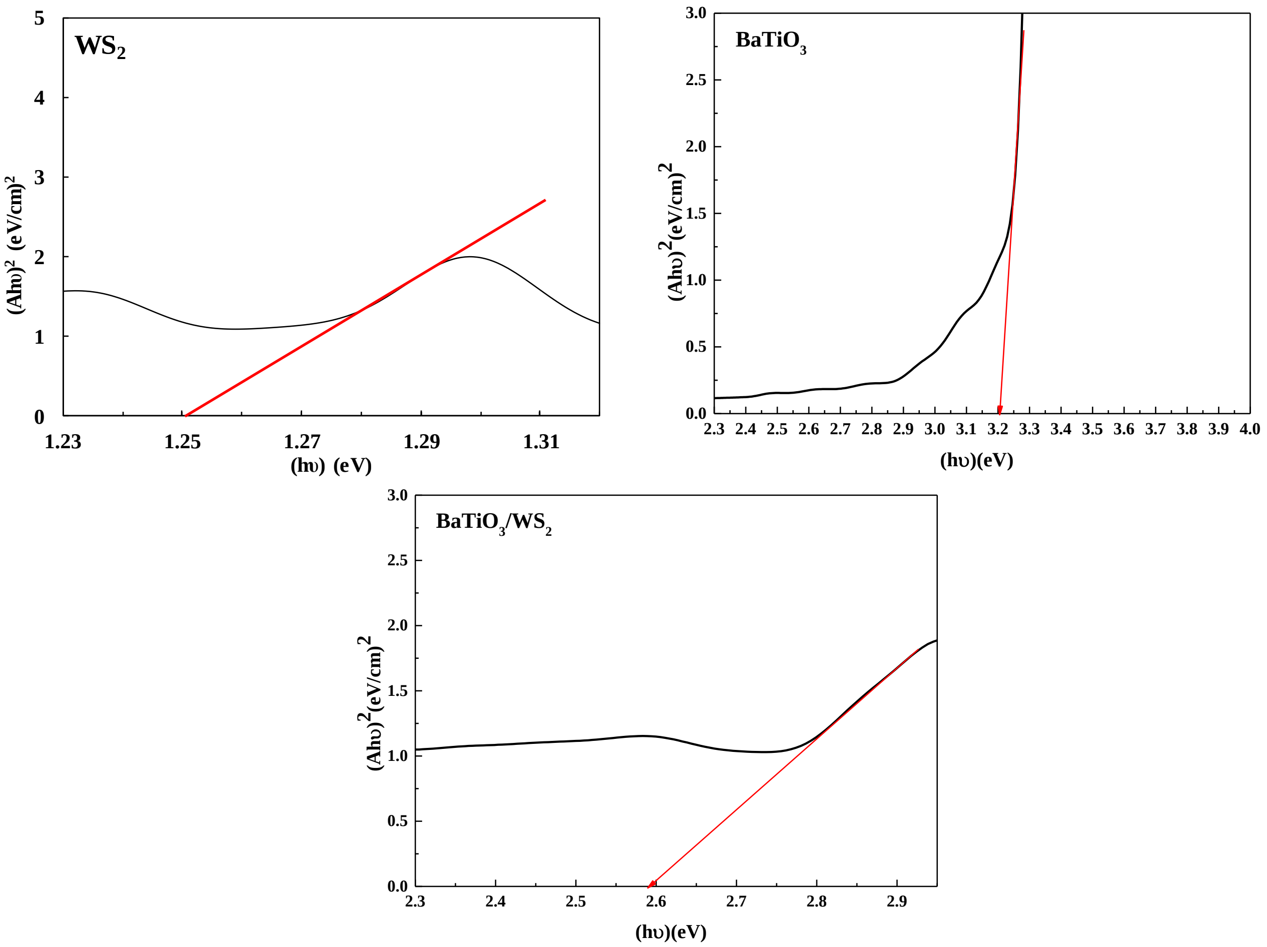

Fig. S5. The impact of different processes on the degradation of OFL.

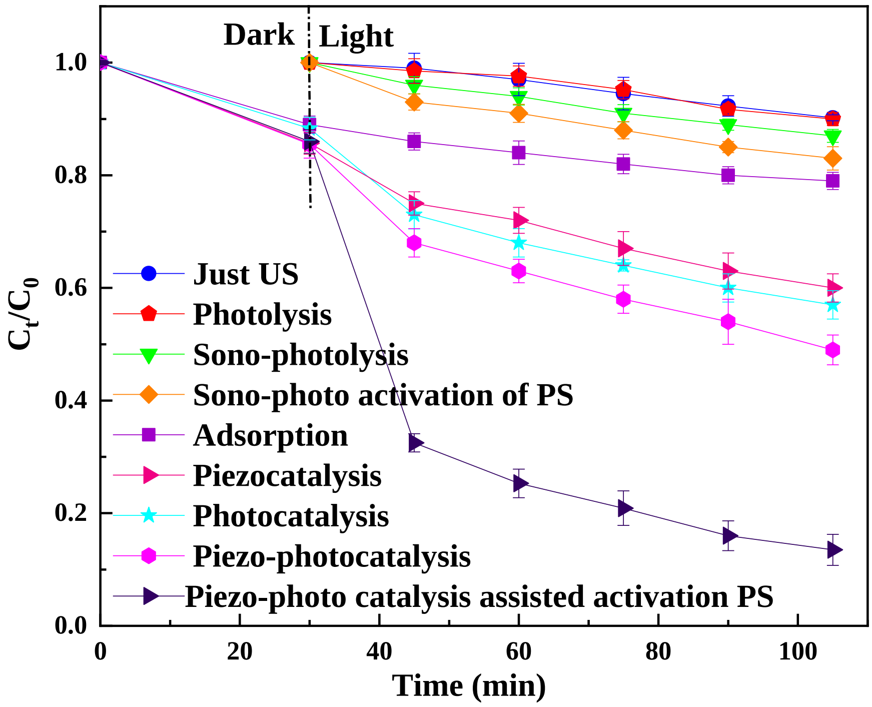

Experimental conditions: Catalyst concentration = 1.0 g L^{-1}, [OFL] = 20 mg L^{-1}, [PS] = 10 mM, pH= 6.5.

Fig. S6. Piezo-photocatalytic degradation of OFL.

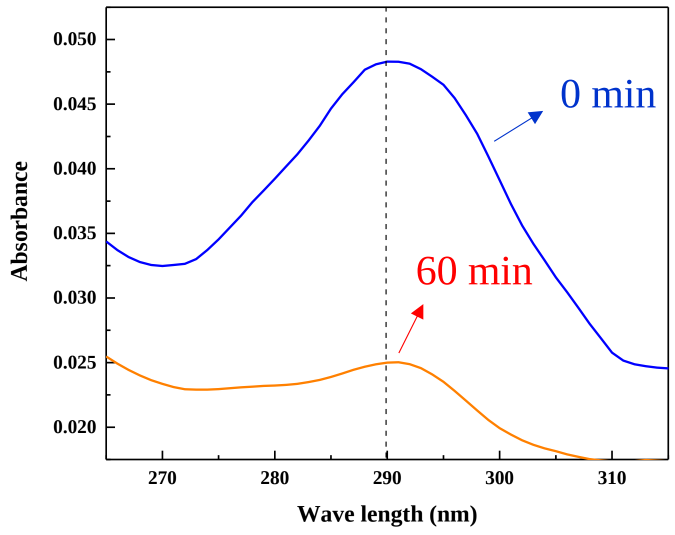

Experimental conditions: Catalyst concentration = 0.025 g L^{-1}, [OFL] = 500 µg L^{-1}, [PS] = 0.25 mM, pH= 6.5.

Fig. S7. Decomposition of PS during the piezo-photocatalytic degradation process.

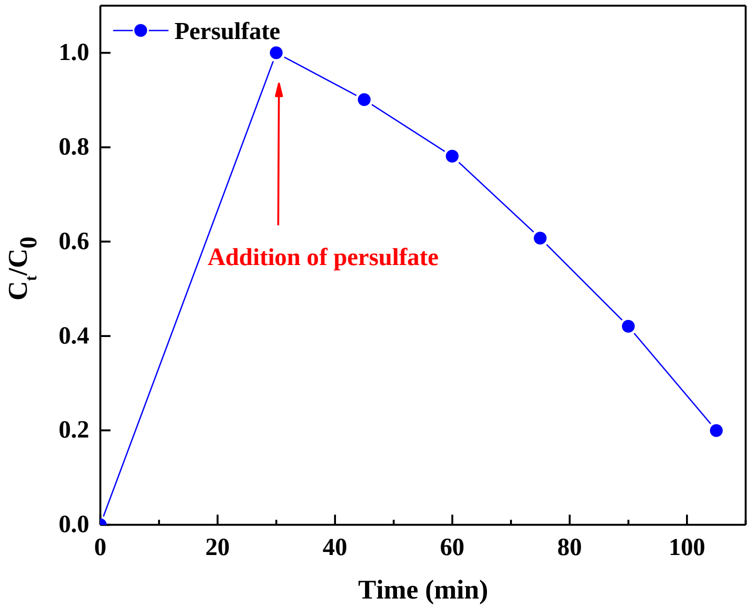

Fig. S8. Degradation of different types of water contaminates during the piezo-photocatalytic activation of PS.

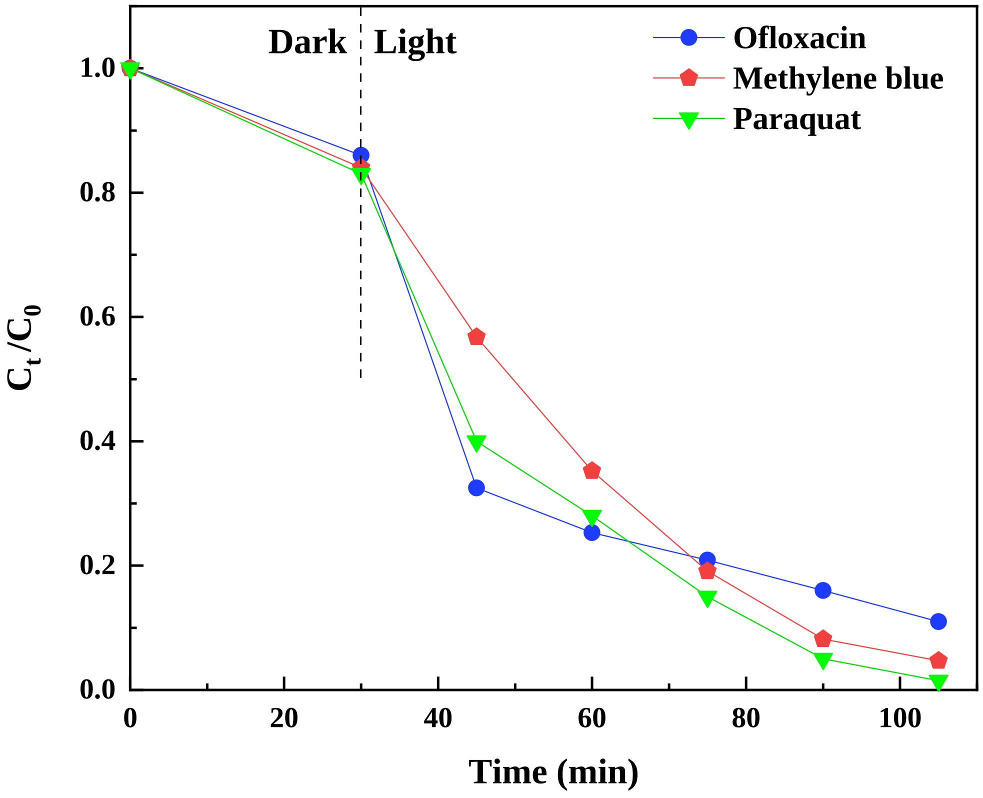

Experimental conditions: Catalyst dosage = 1.0 g L^{-1}, [PS] = 10 mmol L^{-1}, pH= 6.5.

Fig. S9. The XPS valance of WS_2 and $BaTiO_3$.

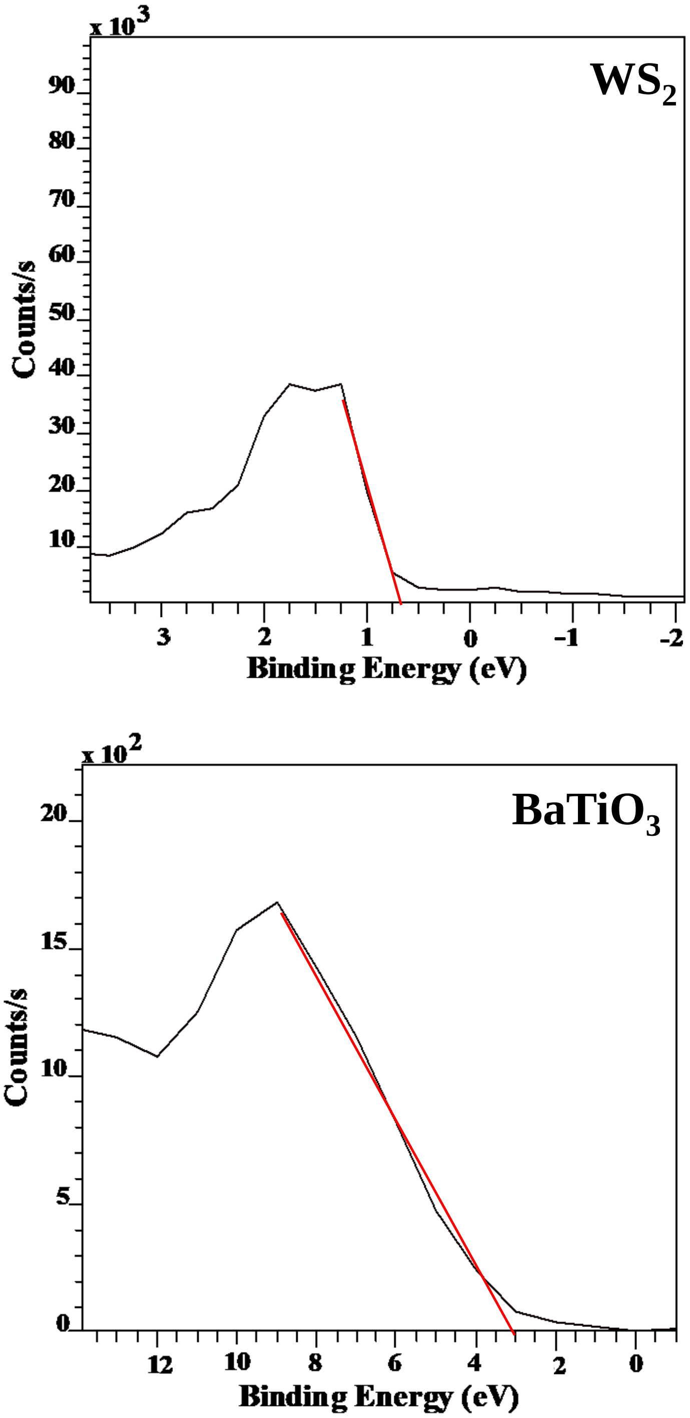

Fig. S10. GC-MS chromatogram of the obtained intermediates during the degradation of OFL.

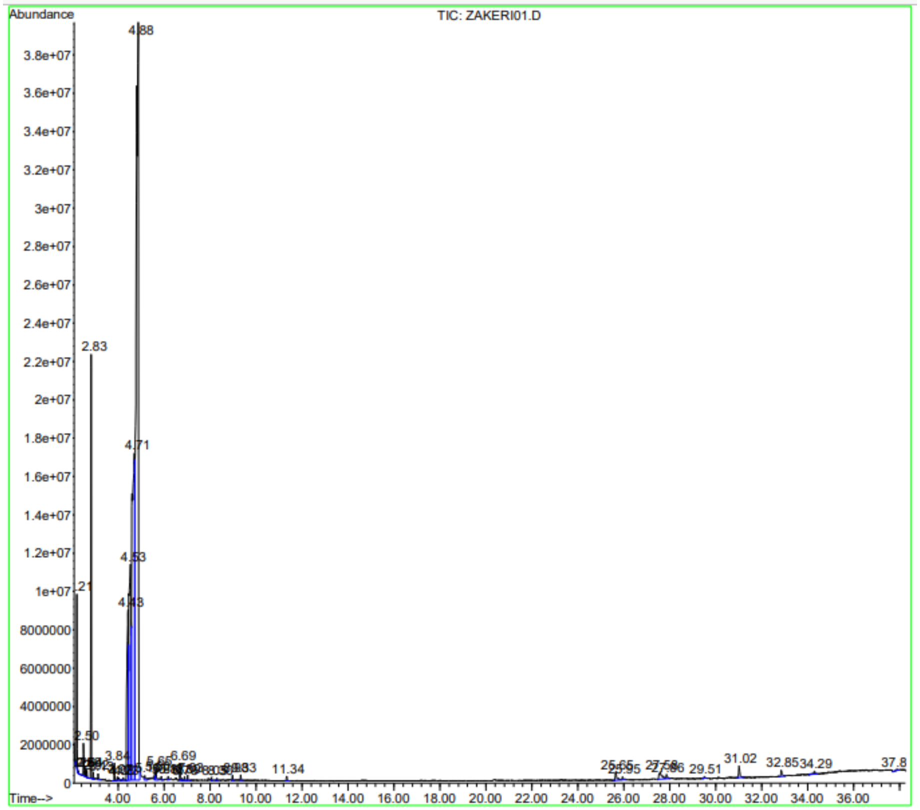

Fig. S11. Schematic synthesis method for the $BaTiO_3/WS_2$ composite.

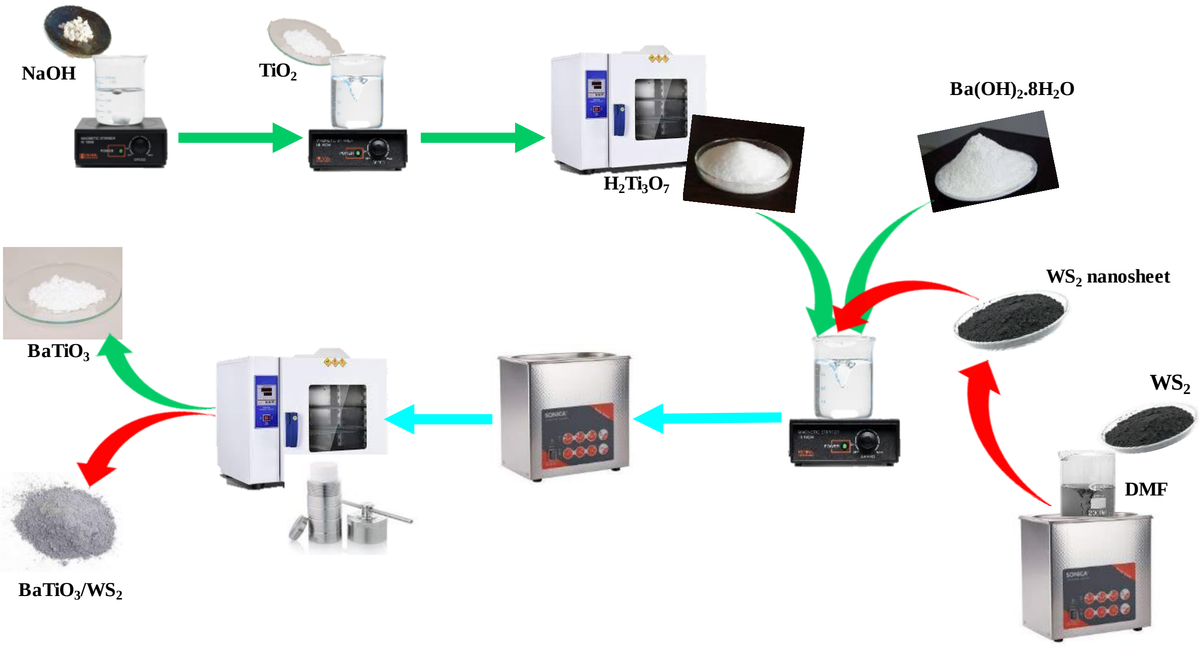

Supplementary Tables

Table.S1. Identified intermediates during degradation of OFL.

NO	Name	Structure	R.T (min)	Main fragments (m/z)	Chromatogram (After derivation)
**1**	(E)-trimethyl (3-methyl-1-phenylpenta-2,4-dien-2-yl)-l4-oxidane	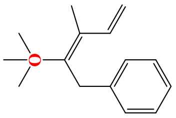	2.640	163.00, 133.00, 75.00, 164.00, 134.00	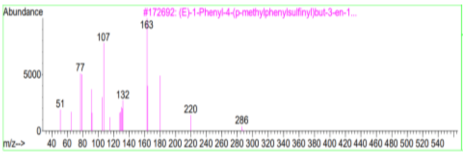
**2**	N, N-diethyl-2-methylpropan-2-amine	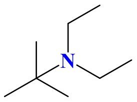	2.929	13.10, 73.10, 75.10, 131.10, 145.10	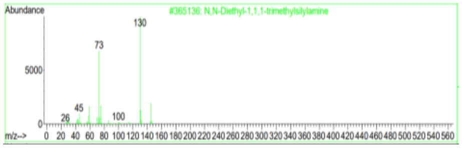
**3**	4,4'-(oxybis (propane-2,2-diyl)) bis (methylbenzene	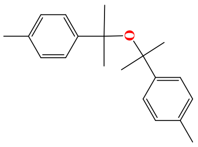	4.027	147.10, 75.10, 73.10, 148.10, 66.10	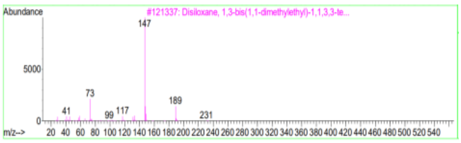
**4**	N-(tert-butyl) acetamide	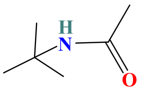	4.438	75.10, 116.10, 73.10, 117.10, 7610	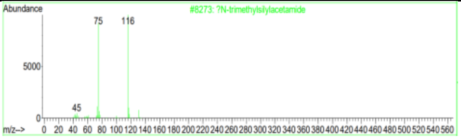
**5**	Acetic acid	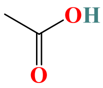	4.49	75.10, 116.10, 73.10, 117.10, 76.10	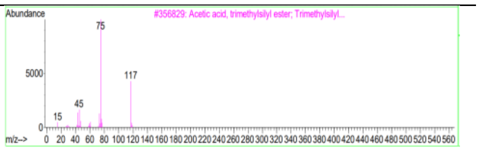
**6**	Ethanimidic acid	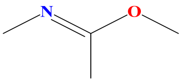	4.870	147.10, 73.10, 203.10, 148.10, 188.10	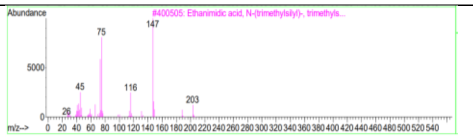

**7**	2-methyl propane-2-ol	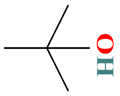	5.603	75.10, 73.10, 130.10, 116.10, 74.10	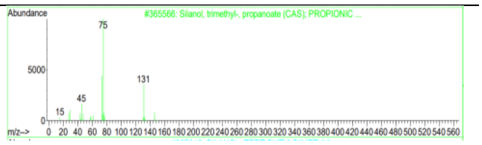
**8**	Methyl (E)-N-methyl acetimidate	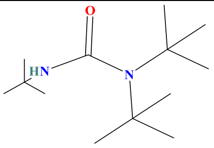	8.976	147.10, 73.10, 75.10, 25.20, 260.10	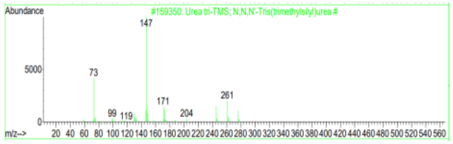

Table S2.Variation of the solution pH before and after the degradation process.

**Solution condition**	**pH value**	**Time (min)**
OFL (20 ppm)	6.5	0
OFL (20 ppm)+ $BaTiO_3/WS_2$ (immediately)	6.57	0
OFL (20 ppm)+_$BaTiO_3/WS_2$ (after 30 min stirring at dark)	6.57	30
Above solution+ PS	6.32	30
15 min (after degradation process)	6.23	45
30 min (after degradation process)	6.11	60
45 min (after degradation process)	6.02	75
60 min (after degradation process)	5.94	90
75 min (after degradation process)	5.81	105
