# Supplementary material for: A BaTiO3/WS2 composite for piezo-photocatalytic persulfate activation and ofloxacin degradation
Source: Commun Chem. 2022 Aug 10;5:95. doi: 10.1038/s42004-022-00707-2 (PMC9814951; doi:10.1038/s42004-022-00707-2)
Supplement: Supplementary file 2 — Description of Additional Supplementary Files [file 42004_2022_707_MOESM2_ESM.docx]

Description of Additional Supplementary Files

**File name:** Supplementary Movie 1

**Description:** Calculation of d-spacing for BaTiO3

**File name:** Supplementary Movie 2

**Description:** Calculation of d-spacing for WS2
